# Tailored Flax-Reinforced Composites: Properties and Sustainable Applications

**DOI:** 10.3390/polym18172069

**Published:** 2026-08-26

**Authors:** Andrei Bencze, Zoran Bergant, Irina Arnăutu, Roman Šturm, Milan Chlada, Rozina Steigmann, Mariana Domnica Stanciu, Adriana Savin

**Affiliations:** 1 Faculty of Mechanical Engineering, Transilvania University of Brașov, 29 Eroilor Blvd., 500036 Brașov, Romania; andrei.bencze@unitbv.ro (A.B.); mariana.stanciu@unitbv.ro (M.D.S.); 2 Faculty of Mechanical Engineering, University of Ljubljana, Aškerčeva Cesta 6, 1000 Ljubljana, Slovenia; roman.sturm@fs.uni-lj.si; 3 Faculty of Industrial Design and Business Management, “Gheorghe Asachi” Technical University of Iași, 29 Prof. Dr. Doc. D. Mangeron Blvd., 700050 Iași, Romania; irina.arnautu@academic.tuiasi.ro; 4 Czech Academy of Sciences, Dolejškova 1402/5, 182 00 Prague, Czech Republic; chlada@it.cas.cz; 5 Institute of Research and Development for Technical Physics, 47 Prof. Dr. Doc. D. Mangeron Blvd., 700050 Iași, Romania; steigmann@phys-iasi.ro

**Keywords:** biocomposites, flax fibers, flax-reinforced composites, sustainability, mechanical properties, dynamic mechanical analysis

## Abstract

Tailored flax-reinforced composites (TFRC) are being investigated as a sustainable alternative to conventional glass- and carbon-fiber-reinforced composites, having a lower fiber volume than these and the structure of the laminate with two plies (two identical layers of alkali-treated flax yarns) is unidirectional, weakly twisted, oriented at ±45°, and reinforced by stitching; it is also impregnated with bio-resin and has robust reinforcement through controlled lamination. TFRC has shear-dominated behavior under both tensile and compressive loading, due to the off-axis orientation of the fibers, but the damage evolution differs significantly. Under tensile loading, the material exhibits a lower strength (55.2 MPa), whereas under compression loading, the composite achieves a higher apparent strength (99.1 MPa). The paper provides a comprehensive analysis for structural and mechanical characterization using the following: nondestructive evaluation using ultrasound to detect internal discontinuities and assess homogeneity; optical microscopy to evaluate fiber–matrix integration and porosity reduction; and Dynamic Mechanical Analysis to assess thermomechanical transitions and storage modulus stability. Finite element simulations have been used to determine elastic properties and validate the matrix-dominated shear response. The results confirm that the [±45°]_4S_ sequences of TFRC optimize mechanical response and interfacial adhesion, promoting TFRC as an ecological solution for structural systems where progressive energy dissipation is preferred.

## 1. Introduction

Over the past decades, research on the properties of natural fibers has fostered interest in their integration into advanced industrial sectors, extending beyond traditional and artisanal textile production. Currently, the industry is taking a step forward by efficiently leveraging the properties of natural fibers in the development of biocomposites, which are materials of the future that use renewable resources with a low environmental impact. Initially used as raw materials for interior design and architectural structures, natural fibers have gradually been incorporated into components in the automotive, sports, and maritime sectors, offering low cost, a pleasant appearance, and high-performance mechanical properties as an alternative to glass fiber-reinforced composites (GFRC). This development builds on the growing interest in biocomposites as materials of the future that use renewable resources with a low environmental impact, and responds to the challenges posed by the unsustainable production of raw materials.

Thus, in recent decades, in line with industry standards, in the production of passenger cars, the trend towards the use of lightweight, durable composites (both for interior components and exterior parts, i.e., the body), which contribute to weight reduction, has led to the use of flax fibers as an ecological alternative to the use of glass/carbon fibers, with direct benefits on hydrocarbon consumption and aging resistance. The abandonment of traditional materials in favor of bio-based ones has required confirmation from existing applications in the field. The selection of biocomposite materials for use and the prediction of their safety in operation over time, based on component analysis, must ensure a technological transfer that integrates with existing manufacturing processes. A 100% ecological biocomposite material consists of a matrix (resin) and a reinforcement of natural fibers [1]. Flax fibers in biocomposites are an alternative to composites reinforced with inorganic fibers (glass, carbon) for semi-structural components, but the relatively low mechanical properties (strength and stiffness) of flax fibers have led to their initial use in hybrid structures (combined with carbon or glass fibers) depending on industrial application [2,3]. Bio-epoxy composites are biocompatible materials, composed of organic or inorganic components [4,5]. The matrix can belong to the group of renewable or non-renewable polymers [6], and plays a major role in protecting the reinforcement from exposure to extreme environmental conditions, including mechanical and/or chemical damage, as well as in supporting loads. In recent years, research on bio-based polymers has increased significantly, closely linked to the disposal of materials after use without harming the environment [7]. Biodegradable polymers can function for a limited period, after which they are converted into products that are easy to utilize further (energy, compost, etc.).

The biodegradable process [8] depends on the composition of the material. Studies carried out on the components of a composite, matrix, and reinforcement aimed to improve the basic mechanical properties and expand their functionality. Natural fibers have gained attention due to their recyclability, low cost, specific strength and stiffness, low density, and potential for chemical modification, which can improve the performance of natural fiber-reinforced composites [9]. Depending on their origin, natural fibers can be classified into vegetable fibers (e.g., cotton, flax, hemp), animal fibers (e.g., wool, silk), and mineral fibers (e.g., asbestos). Natural plant fibers are composed of cellulose, hemicellulose, lignin, pectin, and wax [10,11,12]. In the case of spun yarns, the discrete nature of staple fibers can lead to several modes of fiber migration, depending on external factors [13]. Fiber mobility influences their orientation, cohesion, and mechanical performance during yarn formation and subsequent use in composites. This characteristic is important for evaluating and predicting the properties of natural fiber-reinforced composites [14]. Taking into account the presence of random discontinuities supports the use of nondestructive testing (NDT) techniques such as ultrasound [15], micro-computed tomography [16], C-scan imaging [17], or optical inspection methods [18]. Despite environmental regulations mandating the use of sustainable materials, fiber-reinforced polymer (FRP) continues to dominate sectors such as automotive, construction, aerospace, and consumer goods, underscoring the importance of a comprehensive analysis of its application across diverse structural systems [19]. Whether used alone or in combination with established materials such as fiberglass or carbon composites, natural fibers have been shown to enhance operational performance, providing both reduced weight and superior esthetic quality.

Among the plants commonly used to obtain natural fiber for reinforcing biocomposites, flax and hemp stand out due to their potential to replace glass fibers in certain applications. Flax fibers are derived from the bast tissue of the flax stem and are among the oldest textile fibers known in the world. Flax outperforms many natural fibers, such as cotton, wool, and silk, in both technological processing performance and its ability to enhance product quality and application potential. Although some types of natural fibers possess properties similar to those of man-made fibers and offer greater sustainability [20], they also have some disadvantages. They are more sensitive to humidity and temperature, having the hydroxyl group (OH) that attracts water molecules [21]. They are very dependent on growth conditions (they have high sensitivity to the external environment); they depend on the maturity time and the method of fiber extraction, and the composition (in the case of flax lignin 2–3%) and natural wax can make adhesion to the matrix difficult [22]. The trend is to replace petroleum-based epoxy materials, such as bisphenol A, with polymers derived from natural sources. A disadvantage of biocomposites is the uncertainty in final properties, low thermal stability, and mechanical properties that depend on the materials involved in their production. The natural variability of vegetable fibers, with consequences for specific properties of interest for composite applications (adhesion to the matrix, mechanical performance, tendency to form agglomerates with resins, etc.), requires solving technological manufacturing problems and predicting performance through the development of virtual engineering. The increasing complexity of biocomposite structures and the potential for expanding their use in specialized applications suggest the need to investigate and identify the damage and failure parameters, the causes of their occurrence, and the mode of damage in a composite laminate with a flax fiber fabric [18]. However, unlike artificial fibers, whose properties can be determined unambiguously [23], natural fiber is characterized by a large dispersion of characteristics [24]. By arranging natural fibers (optimized into controllable flax yarns to overcome humidity-induced swelling) within a customized structure, advanced techniques can be used to create efficient geometries for a wide range of industrial applications. The customized arrangement of fibers, in this case tailored flax-reinforced composites (TFRC), with the [+45°/−45°]_4S_ structure directly originates from traditional practices in the textile industry [25]. Because stress distribution in a material under combined loads depends on its mechanical properties, a detailed structural evaluation of TFRC using quantitative and qualitative analyses can enhance the performance of TFRC structural elements. The stacking sequence [±45°/±45°/±45°/±45°] obtained during TFRC manufacturing confers promising properties for specialized applications [26]. The fiber orientation in the aforementioned structure, through its stacking sequences, provides the solid reinforcement necessary for civil engineering/aeronautical infrastructure, where high toughness and progressive damage behavior are preferred over catastrophic failures.

This paper presents a comprehensive qualitative and quantitative analysis (using FEM and experimental results) of customized structures made from flax composite plates with a [+45°/−45°]_4S_ stacking sequence, where each layer consists of two plies sewn together in a [+45°/−45°] configuration using unidirectional (UD) yarns. Testing ± 45° laminates is particularly important because it provides a matrix-dominated in-plane shear response, which is essential for the development and validation of shear-based damage models in composite structures. However, most available studies on ±45° laminates focus on carbon- or glass-fiber-reinforced composites, for example, refs. [27,28,29]. In contrast, investigations of flax fiber composites remain comparatively scarce, particularly regarding their structural failure behavior. Liang et al. [30] investigated the quasi-static response of flax laminates [±45°]_3S_ to characterize in-plane shear properties and compare them with those of E-glass fiber composites. Their results showed that glass fiber composites exhibit significantly higher absolute shear performance, with approximately 43% higher shear modulus and 23% higher in-plane shear strength than flax/epoxy laminates. However, when normalized by density, flax/epoxy achieves nearly equivalent specific in-plane shear strength (≈4% advantage within experimental scatter), highlighting its potential for lightweight applications. Furthermore, Perruchoud et al. [31] investigated the effects of in situ temperature and relative humidity on the monotonic and fatigue response of flax/epoxy laminates ([0/90/0]_S_ and [+45°/−45°]_2S_). The most pronounced environmental sensitivity was observed for the [±45°]_2S_ configuration, where fatigue life decreased by approximately three orders of magnitude under adverse moisture conditions. These findings emphasize that matrix-dominated mechanisms and environmental conditions strongly govern the mechanical response of ±45° flax laminates. Despite these contributions, the [+45°/−45°]_4S_ flax composites remain insufficiently understood, motivating further investigation in the present study.

To detect and locate possible defects (voids, delaminations, fiber non-uniformities in structures) and to inspect the integrity of the adhesive joint, complementary nondestructive evaluation (NDE) methods were employed, including guided waves generated by Hertzian contact, phased-array ultrasound, and optical investigation using microscopy. Dynamic Mechanical Analysis (DMA) was used to measure the thermomechanical transitions of the composites. Finally, the results from numerical simulations were compared with and validated against experimental measurements, ensuring consistency between the modeled and observed mechanical response of the [+45°/−45°]_4S_ flax laminates.

## 2. Materials and Methods

In the last decade, the use of biopolymers as an alternative to petroleum-based plastics has emerged as a solution for global environmental safety. Biodegradable polymers can perform for a limited period, and they can be recycled into products that can be easily further exploited (energy, composting, etc.). This biodegradable process [8] depends on the material composition as well as the type of fibers. The studies carried out on both components of a composite, the matrix and the reinforcement, aimed at improving the basic mechanical properties and expanding their functionality. Composite materials have been engineered to achieve specific mechanical properties when subjected to external loads applied in various ways (tension, compression, or natural agents). Thus, new types of materials have been investigated that combine man-made fibers with natural ones [32,33], or that use natural fibers that are fixed in the fabric with loose polyester or cotton thread, replacing the man-made ones [34,35].

**Figure 1:**
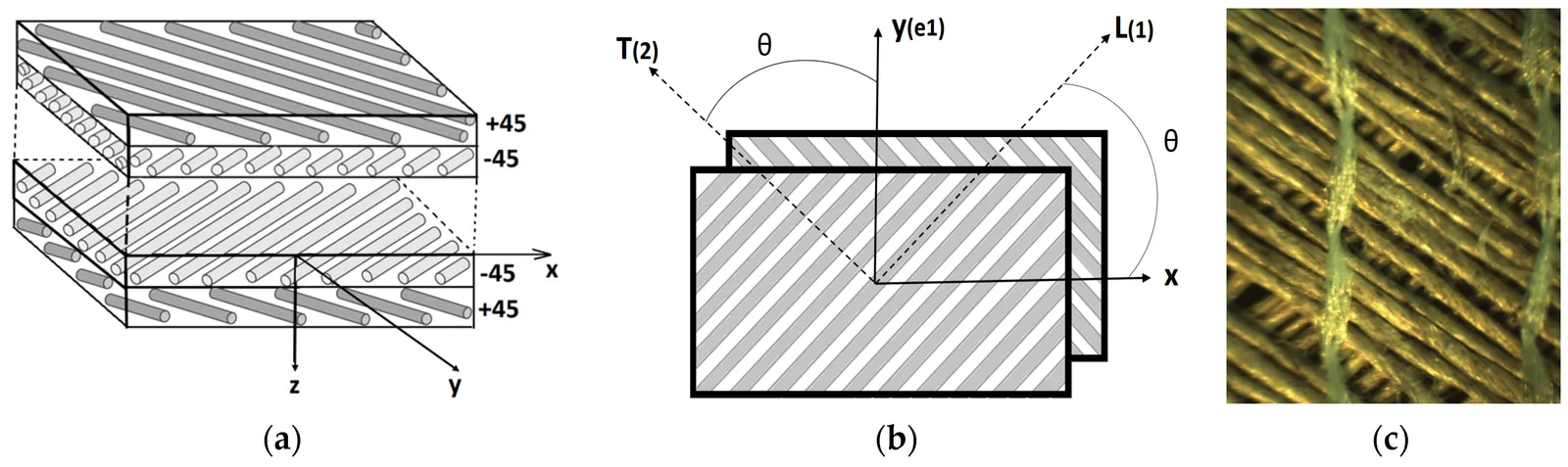
Details of the composite structure: (**a**) symmetric angle-ply laminate [±45°]s; (**b**) off-axis fiber orientations in generally orthotropic lamina; (**c**) two plies stitched together in layer with [+45°/−45°] configuration.

The bio-reinforcement is achieved through an industrial textile technique that stitches together two layers of UD flax yarns, deposited at [+45°/−45°] angles relative to the 0° (e_1_) direction, using a polyester multifilament yarn (Figure 1).

### 2.1. Materials and Samples

Naturally, the two constituents of a composite, the fibers and the resin, significantly different in their structure, make these composites susceptible to various complex damages (delamination, fiber breakage, matrix cracking, etc.). In addition, the load resistance depends on the loading direction with respect to the different stacking sequences [36]. Natural fibers exhibit anisotropic behavior; their mechanical and elastic characteristics greatly influence load resistance when incorporated into a composite, thereby giving it quasi-isotropic (QI) behavior.

**Figure 2:**
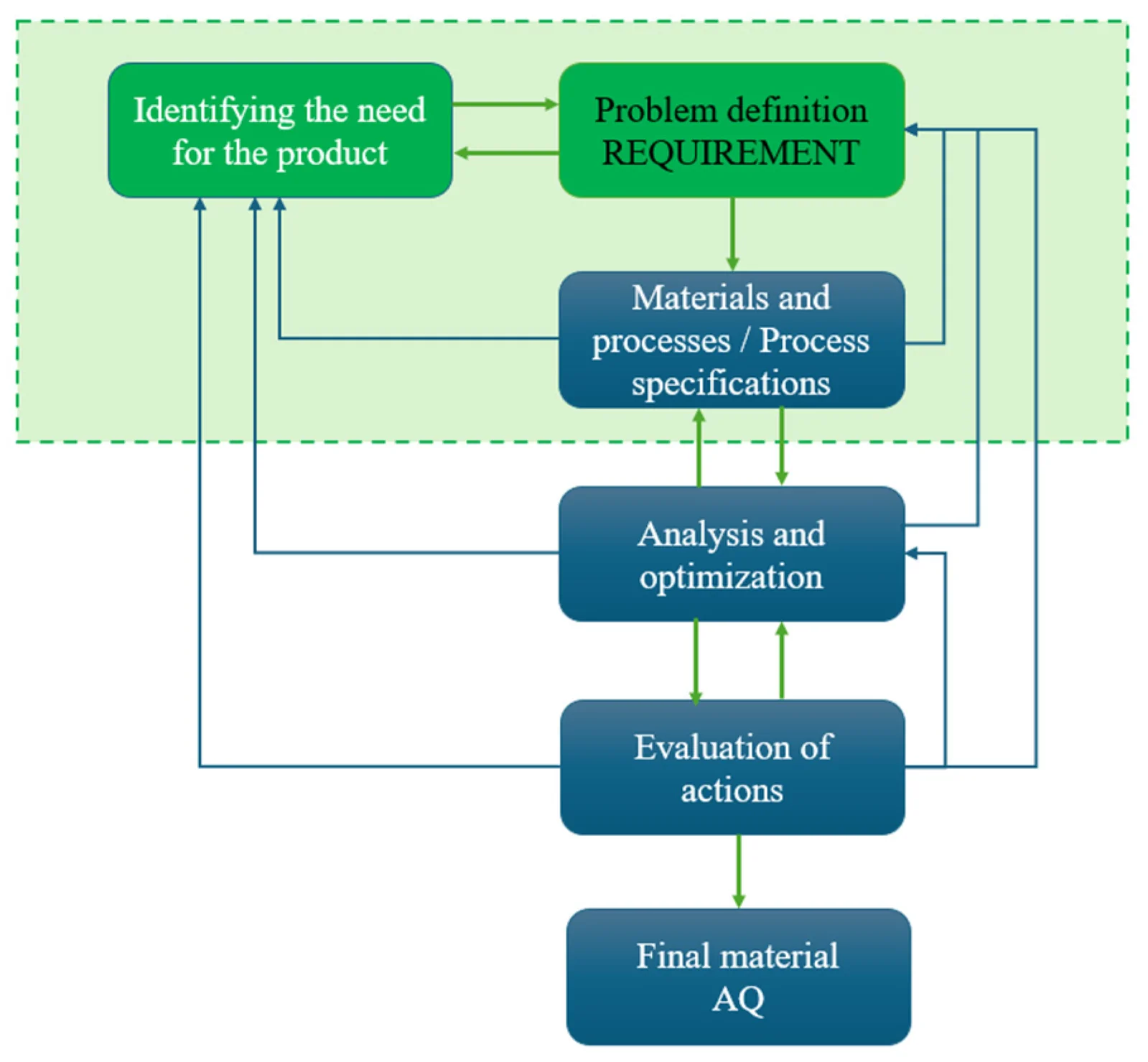
Flow chart of selection criteria for mechanical and physical properties.

The nature of the fibers, the fiber volume fraction (V_f_), and the orientation of the laminate are important factors that influence the mechanical properties, that is to say, the damping of vibrations at some frequencies without modifying the elastic modulus [37]. Theoretical analyses based on calculations and experiments enable the estimation of TFRC characteristics, potentially enhancing mechanical performance in specific applications. The selection procedure for the proposed TFRC was based on key criteria: mechanical properties, physical properties, environmental resistance, manufacturability, and cost (Figure 2).

TFRC has reinforcement made of textile materials in biaxial UD structures. The flax fibers used as reinforcement in TFRC are not conventional technical fibers; they are low-twist yarns that are designed to maximize stiffness and mechanical performance, with good fiber alignment, making them optimal for incorporation into resin. To create stacking sequences from flax yarns, we opted for a textile material with a flat surface measuring 295 × 205 mm^2^, without ripples, and biaxially sewn [+45°/−45°] in an angular sequence of UD yarns. When non-crimp layers made with low-tension yarns are mechanically consolidated by stitching, maximum utilization of fiber properties is achieved [38].

Flax fiber is a natural material that can exhibit some variation. The susceptibility to fiber migration and fragility has been the subject of studies in many works [39,40] which presented the connection between stiffness loss and delamination. The flax yarn layers were reinforced by sewing before resin impregnation, thereby increasing fracture toughness. In ref. [41], It is noted that the load from the stitches, together with their variable spacing, can cause paired breaks in some rows. The flax yarn layers were impregnated with Greenpoxy56 high-performance resin and SD7651 hardener, obtaining a maximum TFRC thickness of 5.38 ± 0.2 mm. The combination ensures a structure with good thermal resistance after post-cure [42] (the SR GreenPoxy56 molecular structure coming from plant origin at almost 56%).

TFRC laminates were manufactured from a biaxial flax fabric in which two laminae oriented at +45° and −45° were stitched together by the manufacturer to form a single manufacturing fabric layer. The fabric consisted of flax yarns with a thread diameter of 0.3178 mm and an areal weight of 223 g/m^2^. Eight biaxial fabric layers, each approximately 0.66 mm thick, were stacked to produce the final laminate, corresponding to a total of 16 plies arranged in the [+45°/−45]_4S_ configuration. The [+45/−45]_4S_ laminate was selected because ±45° plies are primarily responsible for carrying in-plane shear loads. In practical applications, ±45° orientations are widely used in torsionally loaded structures and are typically combined with 0°/90° plies in hybrid laminates to improve damage tolerance and load redistribution. In particular, under torsional loading, ±45°-dominated laminates exhibit significantly higher shear stiffness than 0°/90° layups, making them more effective at resisting shear-driven deformation. Therefore, ±45 layups are commonly used in outer or near-surface layers of aerospace structures such as helicopter rotor blades and wing skins. The fiber volume is V_f_ = 35%, and the density is 1254 kg/m^3^, both lower than those of carbon and glass. The lightweight TFRC was developed to achieve high damage tolerance, necessary to increase the strength-to-weight ratio. The stiffness of the laminate is given by the UD configuration and the minimally twisted fibers. The low-twist yarns and the absence of ripples, as found in fabrics, increase fiber alignment and minimize internal stresses [43], ensuring a higher tensile modulus and reduced deformation. The mechanical and elastic properties of biocomposites depend on the resin, as well as on the nature of the reinforcement, the stacking sequences, and the properties of the laminae. Furthermore, interfacial adhesion influences the mechanical behavior of biocomposites.

#### Analysis of the Flax Fiber-Reinforced Biocomposites

The properties of TFRC depend on several key factors, including resin impregnation and the intrinsic characteristics of the fibers, their pre-industrial processing, and the spinning method used for the flax yarns. Variations in these parameters significantly affect the quality of the fiber–matrix interface and, consequently, the mechanical performance of the TFRC for its intended application.

Flax (Linum usitatissimum) is a lignocellulose fiber that develops as technical fibers (fiber fascicles) composed of multiple elementary flax fibers. The properties of flax fibers depend on the characteristics and structure of elementary flax fibers, their shape and dimensions within the fascicular network, influenced by the stem region from which they originate (base, middle, tip), as well as their chemical composition and the extraction method used to separate them from the stems. The main properties of elementary flax fibers are presented in Table 1.

In general, lignocellulose fibers exhibit greater resistance to the action of chemical agents than cotton. The pectin substances present in flax fibers also serve an impermeabilizing function. Unlike cotton, the pectin substances in flax are not completely removed during finishing processes. Their total removal would result in the transition from technical fiber to individualized elementary flax fiber. Morphologically, an elementary flax fiber consists of the primary, secondary, and tertiary cell walls, as well as the lumen. The primary wall, located at the exterior of the elementary flax fiber, is thin and contains a low amount of cellulose along with non-cellulosic components. It exhibits a microfibrillar structure, with microfibrils inclined at 35–40° to the fiber axis. The secondary wall constitutes the main component of the elementary flax fiber and has the highest cellulose content. The microfibrils in this wall are arranged helically in concentric layers. Their inclination alternates by 10° from one layer to another, giving the elementary flax fiber a high degree of morphological orientation and consequently superior strength. At the center of the elementary flax fiber lies the lumen, surrounded by a well-defined cellulose layer called the tertiary wall.

A fiber fascicle consists of multiple elementary flax fibers that adhere to each other through the middle lamella, which is a complex matrix of hemicelluloses, lignin, pectin substances, minerals, proteins, waxes, pigments, and other components. Multiple fiber fascicles are interconnected along the stem, forming a continuous fibrous network. The longer the elementary flax fiber in a fiber fascicle and the more regular their cross-sectional shape (polygonal), the stronger the connections that hold them together; additionally, the number of elementary flax fibers in the fascicle and the proportion and chemical composition of the middle lamella influence the flax fiber properties. Thus, fibers with higher lignin content are more rigid and strong but less elastic, which constitutes a disadvantage in the technological processing of flax yarns.

The pre-industrial processing of flax fibers consists of separating the fiber fascicles from the non-fibrous components of the plant stems using physical, chemical, biochemical, and mechanical methods. A key stage in this process is the breakdown of pectin in the extra fascicular lamellae, which destroys the bonds between fiber fascicles and enables their extraction from the stem. This stage does not improve the intrinsic quality of elementary flax fiber, but proper process control ensures the preservation of their original properties [46].

**Figure 3:**
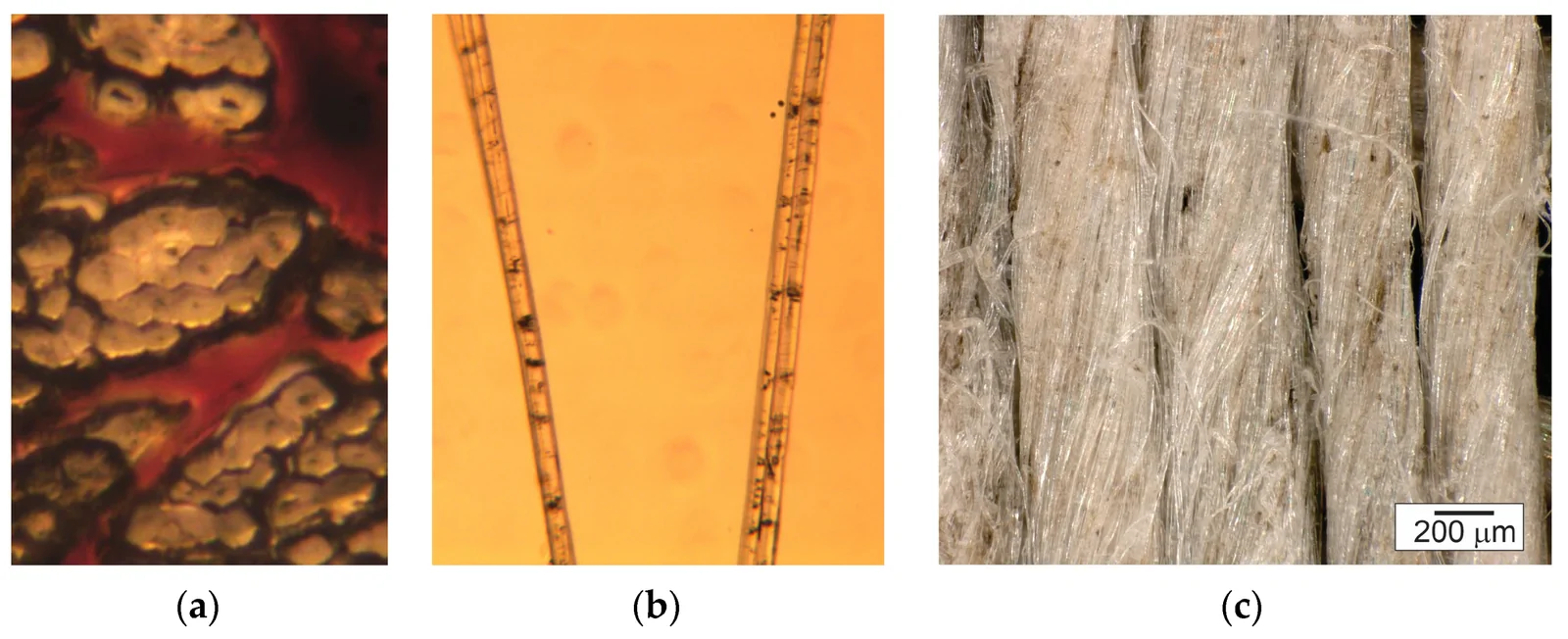
Optical micrograph images of typical flax fiber: (**a**) ×10 cross-sections; (**b**) elementary flax fibers; (**c**) flax yarns.

The microscopic appearance of flax fibers was investigated using optical microscopy techniques (Figure 3). The internal cellular structure was highlighted with a CETI Magnum-T trinocular microscope (CETI^®^ Medline Scientific, Rotherham, UK) for cross-sections (Figure 3a) and elementary flax fiber ×10 (Figure 3b). The OPTIKA SZM-2 stereomicroscope (OPTIKA S.r.l., Ponteranica, Italy) was used to observe the surface morphology of the yarns, as shown in Figure 3c.

Elementary flax fibers can be easily identified under the optical microscope and distinguished from other lignocellulose fibers. In longitudinal view, they are fusiform, with elongated and tapered ends, and exhibit transverse striations resulting from mechanical processing. In cross-section, they exhibit a polygonal shape with rounded corners and a narrow, almost point-like central lumen.

**Figure 4:**
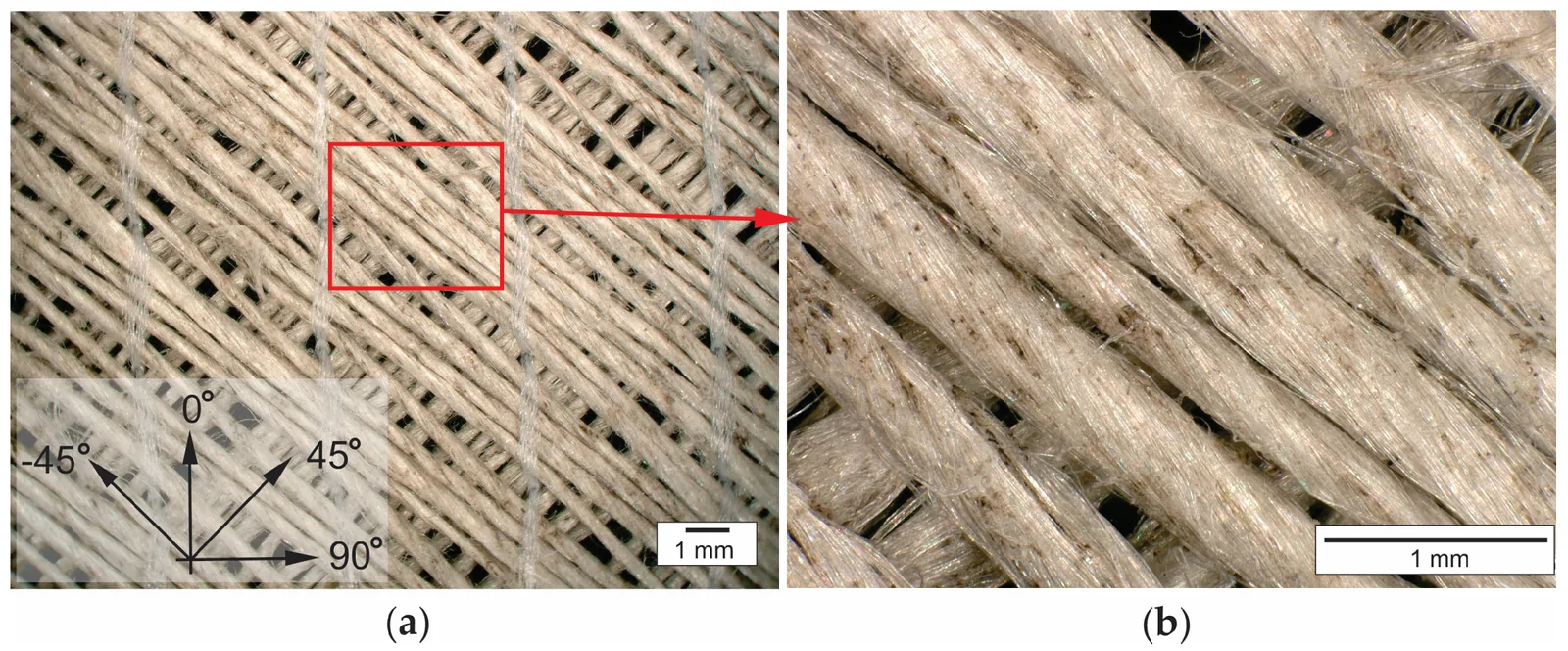
The flax yarn layers from AmpliTex nonwoven fabric: (**a**) 20× magnification (**b**) 100× magnification.

The length of flax fiber fascicles is one of the most important characteristics in the structural design of yarns and the adjustment of spinning machines. The longer and more uniform they are, the higher the quality of the resulting yarns. Figure 4 shows the optical micrograph of the flax biaxial fabric architecture with fiber orientation of ±45°. The image shows the surface morphology of the AmpliTex flax reinforcement (Bcomp, Fribourg, Switzerland), consisting of yarn bundles aligned in two principal directions to form a balanced biaxial structure. The unidirectional flax layers are stabilized by stitching with multifilament polyester yarns. Although the stitches are placed at regular intervals, local irregularities in yarn grouping are visible due to variations in flax fiber bundle thickness. Slight twisting of the long flax fiber fascicles is also evident, indicating the consolidation of elementary fibers into yarns.

Analysis of the characteristics of flax yarns from the layers of the AmpliTex nonwoven fabric used for TFRC highlights a wet-spun, medium-fineness yarn structure, obtained from chemically treated long fibers, which are designed for technical applications. The main structural characteristics of the flax yarns analyzed in this study are presented in Table 2 as average values, both in the dry state for yarns extracted from AmpliTex nonwoven fabric and for yarns embedded in the matrix.

The linear density of the dry yarns was determined using the gravimetric method, and the area of the impregnated yarns was measured through image analysis using Optika ProView digital camera software [47].

In Table 2, the yarn twist is calculated using Köchlin’s relationship as a function of yarn count (*Nm*) and twist coefficient (α*_m_*): $T=\alpha_{m}\cdot \sqrt{Nm}$ [twists·m^−1^] and diameter of the yarn, $D=C/\sqrt{Nm}$ [mm], where *C* is the flax yarn constant. The conversion between yarn count (*Nm*) and linear density (*T_tex_*) is *T_tex_* = 10^3^*/Nm*, where *T_tex_* is expressed in [g/km].

A significant reduction in yarn linear density (approximately 57%, from 100 tex to 43 tex) is observed after embedding in the matrix, attributable to structural compaction. In the transverse section through TFRC, the overlapping flax yarn layers generally maintain their alignment, intercalating slightly in places, while the epoxy resin fills the voids. This provides yarn reinforcement and can delay fracture under conditions of imminent impact.

**Figure 5:**
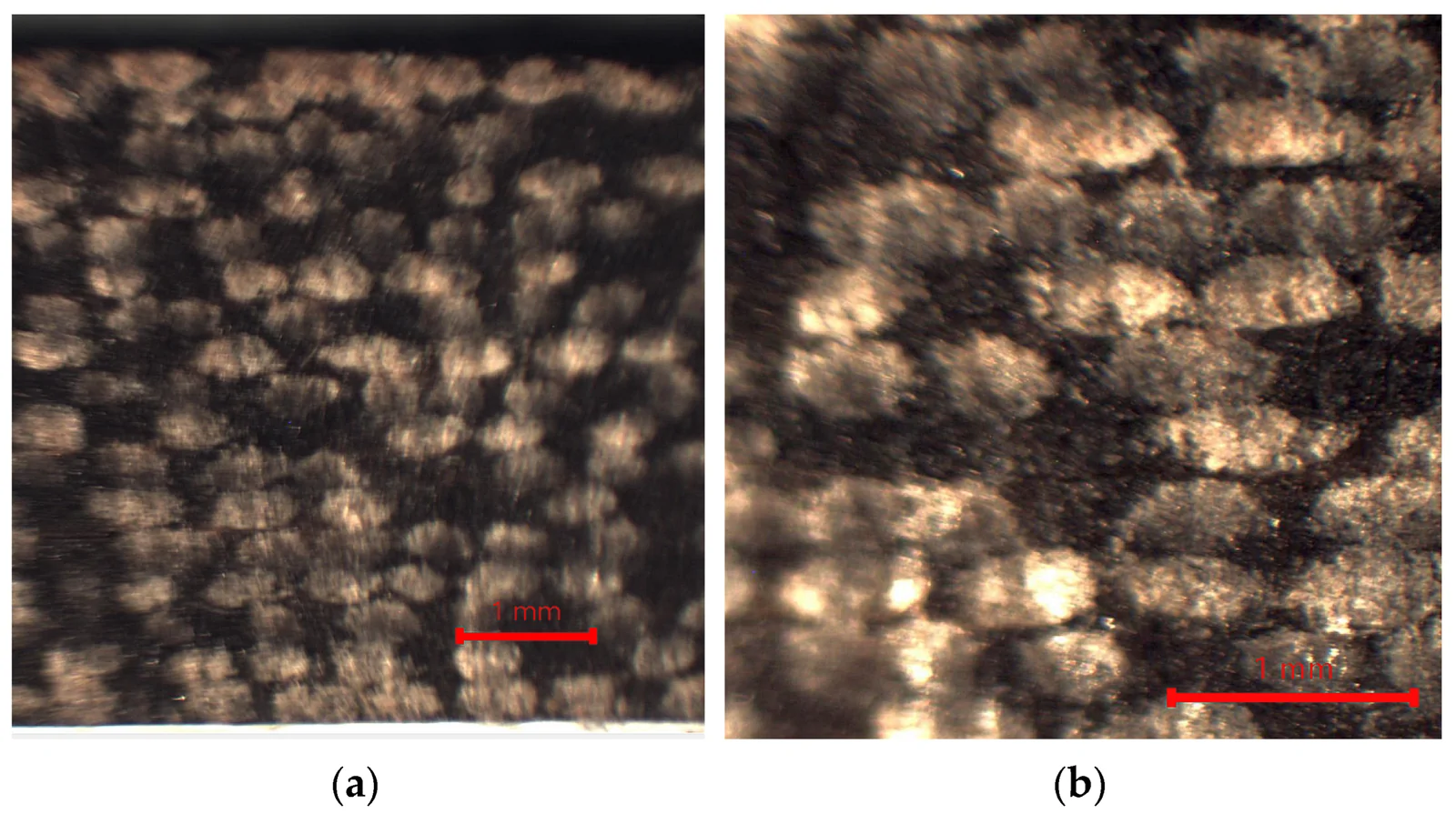
Cross-sectional morphology of the [+45°/−45°]_4S_ TFRC laminate (**a**) cross-section of 16 ply laminate; (**b**) detailed view of yarns.

The yarn cross-sections exhibit irregular, oval-shaped contours with a compact structure. The TFRC, observed under an OPTIKA SZM–2 stereomicroscope, exhibits a dense, uniform, and lightweight appearance (Figure 5).

In addition to fiber orientation within yarns, the mechanical performance of TFRCs is strongly influenced by surface treatments that enhance fiber–matrix adhesion [48].

The removal of impurities is carried out by alkaline treatment of the fiber sliver or roving, before the wet spinning of flax yarns. The acceptable limits of the alkaline treatment are associated with minimizing the degradation of elementary flax fibers and involve, especially, the use of a low-concentration sodium hydroxide solution and a shorter processing time than in cotton fiber mercerization. Its role is to partially dissolve lignin, hemicelluloses, and pectin substances in the intercellular regions, which act as natural binders between the elementary flax fibers, as well as to remove waxes and fats from the surface of the fibers.

The alkali-treated yarns exhibit high compactness, increased hydrophilicity, and improved fiber mechanical properties. Following their incorporation and compaction into the epoxy resin matrix, the mechanical properties of the elementary fibers will be transferred to a higher degree to the composite material, thereby contributing to its mechanical behavior [49].

**Figure 6:**
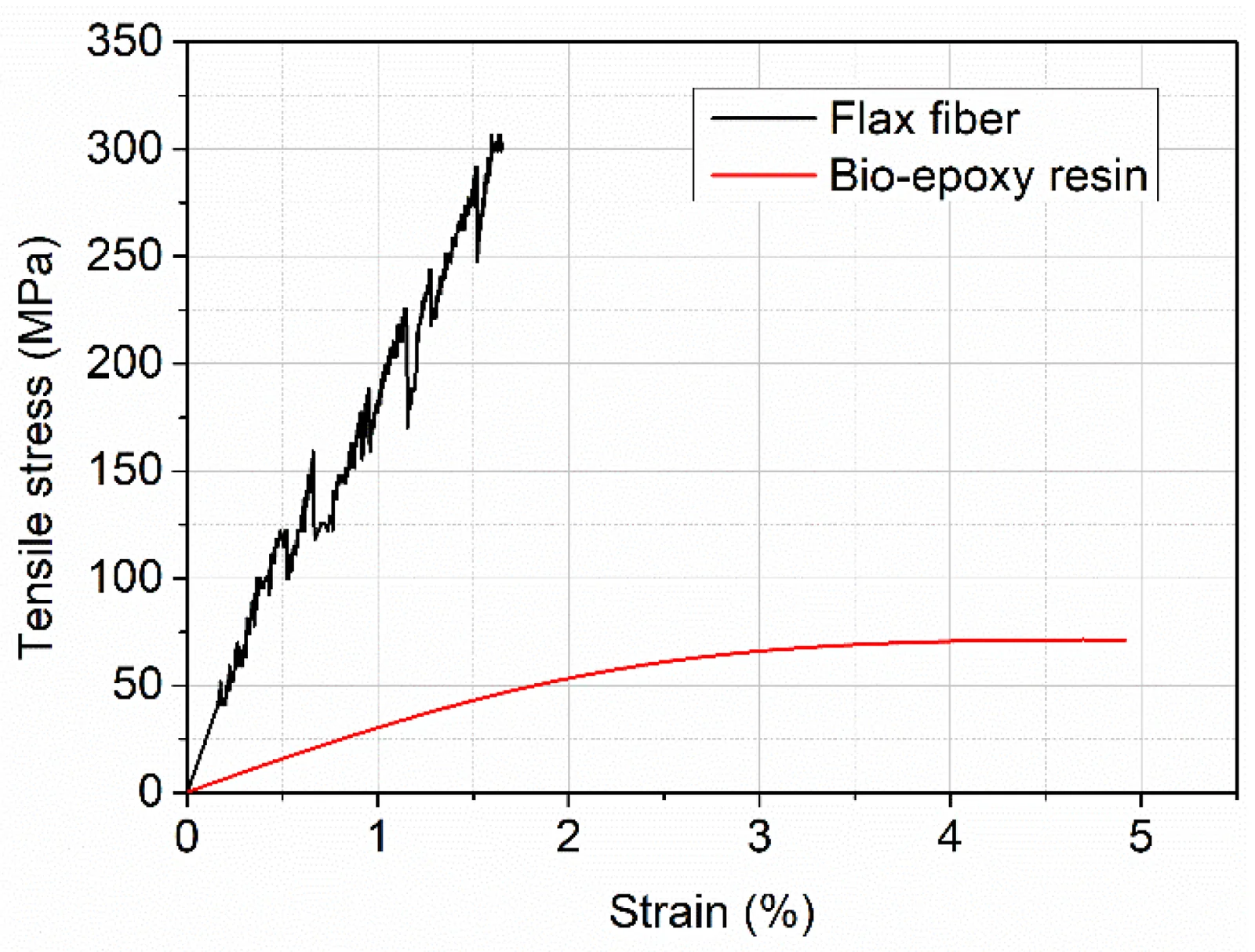
Stress–strain for non-impregnated yarns and epoxy matrix.

The tested flax yarns were evaluated in a non-impregnated state in tension. Due to its twisted structure and porosity, the load is not uniformly distributed among the elementary fibrils. During tensile loading, the fiber undergoes an initial straightening phase, followed by progressive slippage and the local failure of individual constituents. This results in a characteristic step-like stress/strain response, where multiple small load drops occur before final fracture. The Young’s modulus was estimated from the initial linear portion of the stress–strain curves. The flax yarn exhibited an effective modulus of *E_f_* = 21.43 GPa, while the bio-epoxy matrix showed *E_m_* = 3.23 GPa. The relatively low modulus of the flax yarn, compared to values reported for elementary flax fibers, is attributed to the twisted structure, porosity, and progressive slippage between fibrils (Figure 6).

The Young’s modulus of both constituents was estimated from the initial linear region of the stress–strain curves. The flax yarn exhibited an effective modulus of *E_f_* = 21.43 GPa, while the bio-epoxy matrix showed a modulus of *E_m_* = 3.23 GPa.

Table 3 shows the average breaking force and tensile strength of flax yarns and epoxy resin.

### 2.2. Methods

#### 2.2.1. Ultrasound Test

The interaction of TFRC with the ultrasound beam (US) depends on the US energy but also on the structure of the composite, i.e., the dimensions and cohesion of the fibers, the quality and composition of the bio-epoxy resin, the moisture content, the environmental temperature, and, especially, the orientation of the packing sequences in relation to the US source. The method of analyzing the structure of the TFRC laminate using guided waves generated by Hertzian contact has the advantage of not requiring coupling, and the contact ensures the natural point for Lamb waves. The theoretical problem of Hertzian contact was solved in [50]. In the absence of coupling, the situation where the deformation is transient and can be utilized for US generation and propagation is described in [51].

**Figure 7:**
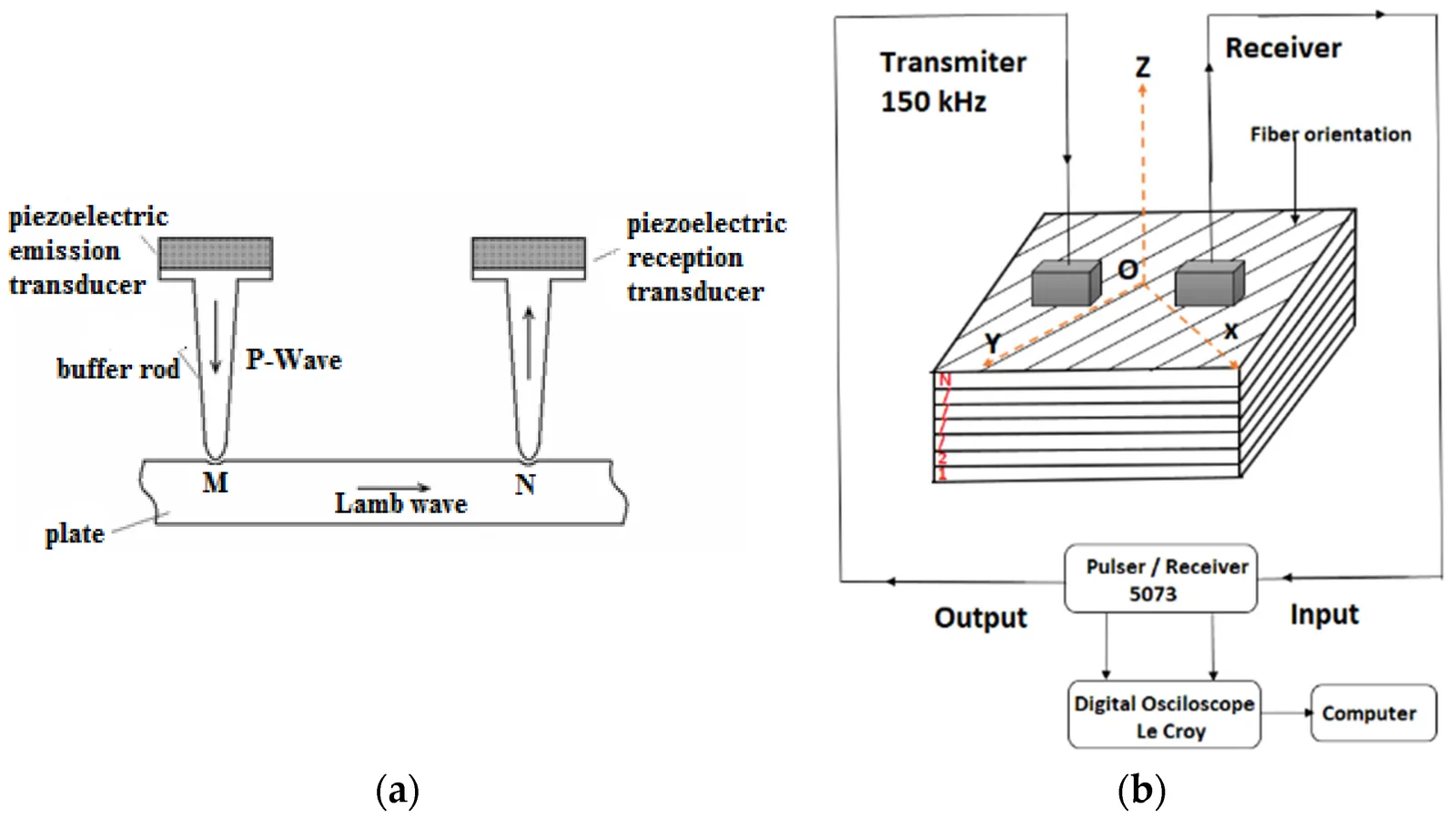
Generation and reception of Lamb waves: (**a**) Hertzian contact transducers; (**b**) diagram of transducer connection to the measuring equipment.

Guided US waves travel as Lamb waves (vertically polarized) and shear waves (horizontally polarized). For composites, in practice, due to high damping, investigations are performed below the cutoff frequency (equal to the product of the frequency and the thickness) of the higher modes. If the distance between the Em–Re peaks of the Hertzian contact transducers is small, both the A0–antisymmetric mode and the S0–symmetric mode are visible [52]. In the case of larger distances (in this case 140 mm), the S0 mode, which is much more dispersive, is practically completely attenuated, with only the A0 mode still propagating [53]. To generate Lamb waves in TFRC, P111-01-P3.1 Introscope transducers were used, with each transducer having a piezoelectric body and a central frequency of 100 kHz, and coupled to an AISI 316L steel buffer rod with a spherical head of 3 mm radius. The method using two Hertzian contact transducers connected in a send–receive system and coupled to an ultrasonic Pulse Transmitter/Receiver is presented in [54]. The diagram showing how to connect the transducers to the measuring equipment, with the transducers placed on the laminate, is shown in Figure 7. The flax fibers have a thread length that can accommodate an A4-format TFRC plate, avoiding reflections at the ends during measurements.

For a TFRC plate with small thickness, waves were generated and received by transducers coupled to a Pulser–Receiver 5073 PR (Panametrics, NDT, Billerica, MA, USA) connected to a digital oscilloscope, Wave Runner 64Xi (LeCroy, Chestnut Ridge, NY, USA), which allows time measurement with an accuracy of 0.1 ns. The method is used to determine Poisson’s ratio and the phase velocity of waves at 100 kHz, and it allows the study of the elastic properties of TFRC components, as well as the qualitative inspection of the biocomposite. The time-of-flight method (US TOF) is the common technique in US testing. From the time interval between transmission and reception, the wave speed is obtained C=2L/TOF=2Lυ, where ν is the wave frequency [55]. The effect of the percentage of fibers (V_f_) in the composite mass and the fiber orientation correlates with the longitudinal wave velocity of the US as well as with the attenuation of the US wave [56]. The absorption mechanism in woven structures differs from that in nonwovens due to pore geometry and frequency. US waves in porous materials, through channels or interstices, as well as through air molecules that oscillate under the pressure of the exciting US wave, are attenuated. The thicker the composite, the greater the attenuation is. The attenuation coefficient of US in the material is specified by the relation $\alpha =\frac{20\log\left(A/A^{\prime }\right)}{2h}$ where A and A′ represent the average amplitudes of the signal obtained when scanning the surface of the sample (A-scan), and h is the thickness of the plate. Taking into account the mixing rule, the elasticity characteristics will depend on the fiber volume V_f_. To highlight any possible delamination discontinuities or regions of porosity in the material, the Phasor XS equipment (produced by General Electric—Inspection Technology, Skaneateles, NY, USA), coupled with a US transducer with a linear area of 32 and operating at 5 MHz, was used. The transducer was placed on a wedge with an angle of 36°. Linear movement was provided by mounting the transducer on an encoder displacement device, enabling a linear scan of the area of interest.

#### 2.2.2. Tensile and Compressive Static Mechanical Tests

**Figure 8:**
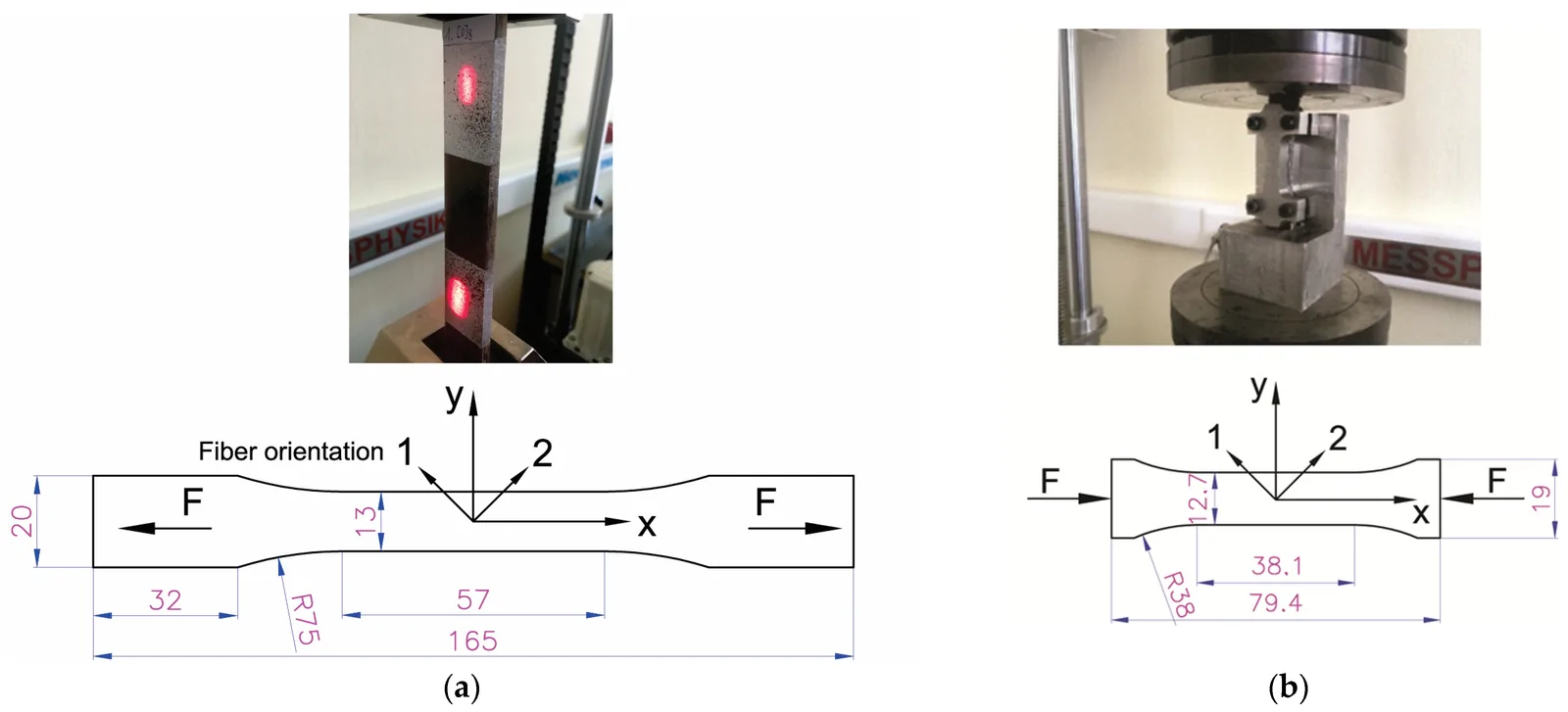
Tensile and compressive test: (**a**) tensile test and specimen geometry (ASTM D638 standard); (**b**) compression test and specimen geometry (ASTM D695 standard).

Specimens were prepared in accordance with ASTM D638 [57] for tensile testing and ASTM D695 [58] for compression testing, and tested on a Messphysik BETA-50 universal testing machine (Messphysik Materials Testing GmbH, Fürstenfeld, Austria). Tensile strains were recorded with an optical laser speckle extensometer (measurement error ± 0.02%) to measure longitudinal strain, with data acquired using a computer-based data acquisition system. In compression tests, the crosshead stroke was monitored. The loading rate was 2 mm/min. The mechanical parameters (Young’s modulus, tensile strength, deformation) were obtained as the average of the values from tests performed on TFRC samples. Each tensile sample is provided with 2.5 mm thick glass fiber tabs at the gripping region. The compression specimens were designed with widened ends to prevent premature failure due to edge effects during testing. The geometry of the tensile and compressive samples is given in Figure 8.

#### 2.2.3. Dynamic Mechanical Analyzer (DMA)

One of the basic methods for investigating molecular motion in biocomposites is studying the temperature dependence of parameters that characterize dynamic viscoelastic properties. DMA analysis is a well-established technique for measuring the thermomechanical transitions of composites. The testing equipment was a DMA242C (Netzsch, Selb, Germany), with a three-point bending test performed using Protheus software v.4.8.5 under sinusoidal cyclic loading. TFRC specimens for the DMA test were prepared in accordance with the ASTM D5023 procedure [59]. For the analysis of elastic moduli, samples of TFRC with dimensions of 50 × 10 × 5 mm were cut using the OMAX2652A machine (OMAX Corporation, Seattle, WA, USA) along the direction (e_1_) and in the direction inclined by 15° to the e_1_ axis. The measurement conditions were three-point bending test, in the temperature range (27 ÷ 220 °C), at all equipment frequencies [150] Hz, with a force amplitude of 6 N and a maximum deflection of 30 μm. Vf and the stacking sequence strongly influence the strength and elastic modulus of TFRCs. The viscoelastic properties of the samples (complex modulus of elasticity E* (Young modulus) and G* (Coulomb modulus), loss modulus E″, which represents the viscous component of the material, and loss factor tg δ as a function of temperature were determined in the specified temperature range. The parameters obtained from the tests provide information on vitrification, referred to as Tg (glass transition), resulting from the cross-linking reaction. From experiments performed across the temperature range and for all equipment frequencies, two test frequencies, 1 Hz and 5 Hz, were selected to determine the apparent activation energy for the glass transition process.

#### 2.2.4. Finite Element Analysis (FEA)

A finite element (FE) model of the test coupons was developed using MSC Patran and analyzed with MSC Nastran (version 2019). The numerical analysis was performed to support the experimental tensile and compressive characterization of the laminates and to investigate the local stress, strain, and deformation fields within the gauge section of the specimens. The coupons were modeled as shell surfaces with dimensions as shown in Figure 8. A consistent system of units ([N], [mm], [MPa]) was used.

**Figure 9:**
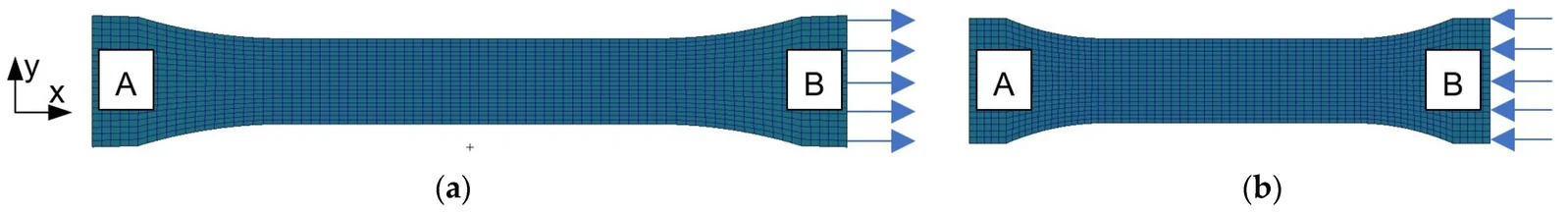
FE model boundary conditions: (**a**) tension coupon; (**b**) compression coupon.

Both tensile and compressive specimens were discretized using four-node quadrilateral (QUAD4) elements (Figure 9). The material was modeled as a linear-elastic, 2D orthotropic lamina using experimentally determined properties. The principal direction of the composite material (0°) was aligned with the sample’s longitudinal axis. The laminate was modeled as a symmetric stacking sequence of 16 plies with the configuration (+45°/−45°)_4S_, with each ply having a thickness of 0.335 mm. Boundary conditions for both tension and compression were defined to replicate the experimental setups. Side A was fully constrained (all six degrees of freedom—DOFs 1 to 6 blocked).

In contrast, side B is constrained in all degrees of freedom except longitudinal translation (DOF 1 free), allowing load application along the specimen longitudinal axis (Figure 9). For the compression model, out-of-plane displacement (DOF 3) was additionally restricted on all nodes to suppress global buckling, consistent with the mechanical test fixture conditions. The load was applied as a uniformly distributed force along the nodes at side B. The applied forces (selected to be within the linear-elastic range of the stress–strain curve) are +2500 N for tension (at strain = 0.8%) and −2500 N for compression (at strain = 1.1%), according to the experimental results presented in Section 3. Linear static analysis (Solution 101 in MSC Nastran) was performed. The linear-elastic FEA was adopted because most composite structural applications (particularly in aerospace or automotive) are designed to ensure that operational loads remain below the onset of damage. Therefore, the material is assumed to remain within the linear-elastic regime, and no permanent deformation occurs. The objective of the analysis is to evaluate stiffness, deformation, and stress–strain distribution, rather than to simulate damage initiation or the progression of failure. The flax composite material is therefore modeled as an orthotropic linear-elastic laminate according to classical laminate theory (CLT). This modeling strategy provides accurate estimates of the global behavior with a reduced computational effort.

## 3. Results and Discussions

### 3.1. Ultrasound Results

**Figure 10:**
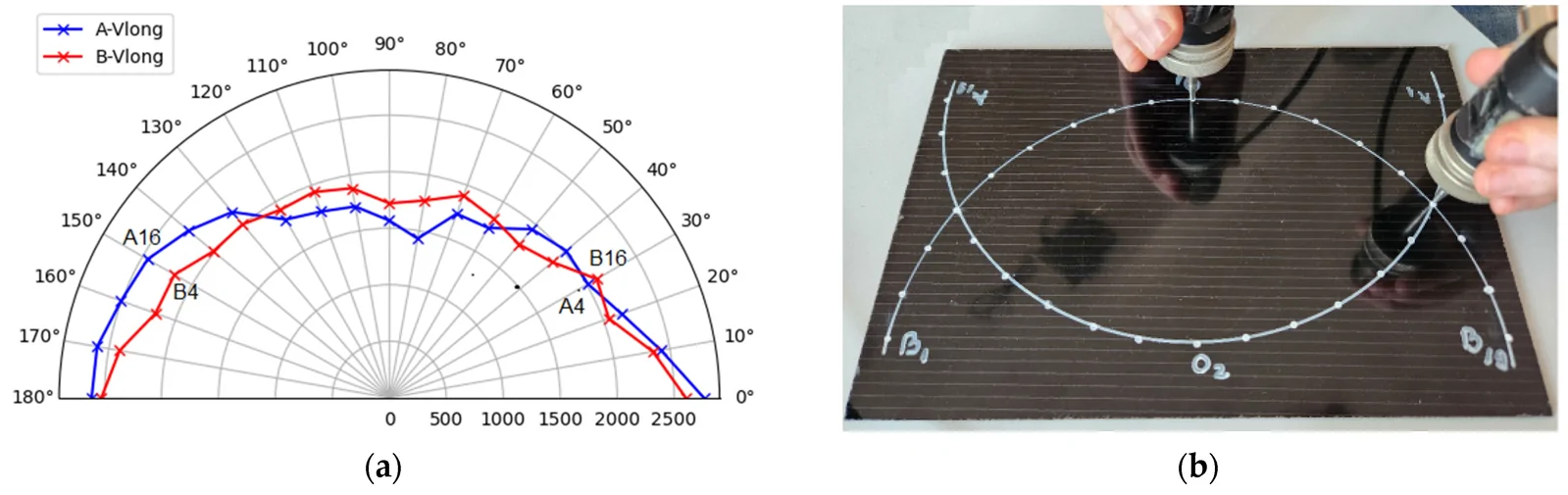
Ultrasound velocity: (**a**) distribution of the group velocity of A0 mode in the studied sample; (**b**) position of measurement sensors in determining the velocities.

The distribution of fibers in the TFRC structure causes multiple scattering of the ultrasonic wave, separating it into direct and diffuse components. In US inspections of flax biocomposites, the frequency is typically reduced to mitigate attenuation and scattering. The Lamb wave method and ultrasonic S-scan (sectorial scan) techniques significantly improve the efficiency of evaluating thin composite materials (such as flax biocomposites) by providing rapid, large-area coverage. These methods are particularly effective for detecting, locating, and quantifying structural anomalies such as delamination, matrix cracking, and impact damage that are difficult to identify with traditional techniques. Guided waves generated by Hertzian contact are more suited for defect screening. This method uses Lamb waves and enables the quick inspection of a wide area of adhesive joints. By applying a force F on the transducer, the buffer metal rod (having the modulus of elasticity E_1_ and Poisson’s ratio ν_1_) produces an elastic deformation of radius a (called Hertzian contact) [60] on the surface of the material (points M and N in Figure 7a). The generation and propagation of Lamb waves are obtained by placing the source (Em) at the center of O1 and receiving the signals at points on the circle arc A1_A19 (marked from 10° to 10°) by sweeping the receiving transducer (Re) along the arc (Figure 10b). The method is applied to the B1_B19 arc, with the source placed at the center of O_2_; the TFRC structure is investigated. Figure 10a shows the group velocity distribution for the A0 mode along the analyzed propagation directions; the QI character of the composite, as indicated by the reinforcement structure, is evident.

A variation in propagation velocities is observed even for the same measurement points (A4; B16) and (A16; B4), respectively, due to the distribution of fibers in the stacking sequence [±45°]_4S_ in the TFRC realization (by changing the Em position in O_2_, the propagation of the ultrasound encounters a different layer structure). The group velocity according to [61] for mode A_0_ is determined from the equation:

(1) $$C_{Ao}={\left(\frac{D}{\rho h}\right)}^{1/4}\omega^{1/2}$$

where *h* is the thickness of the plate, *ρ* is the density, *ω* is the angular frequency, and *D* is the flexural rigidity; knowing the values of *ρ, h, ω*, and the velocity, the *C_A0_* value of *D* can be determined from Equation (1), and then *E* and *ν* in the direction of the Lamb waves for the examined material are determined.

**Figure 11:**
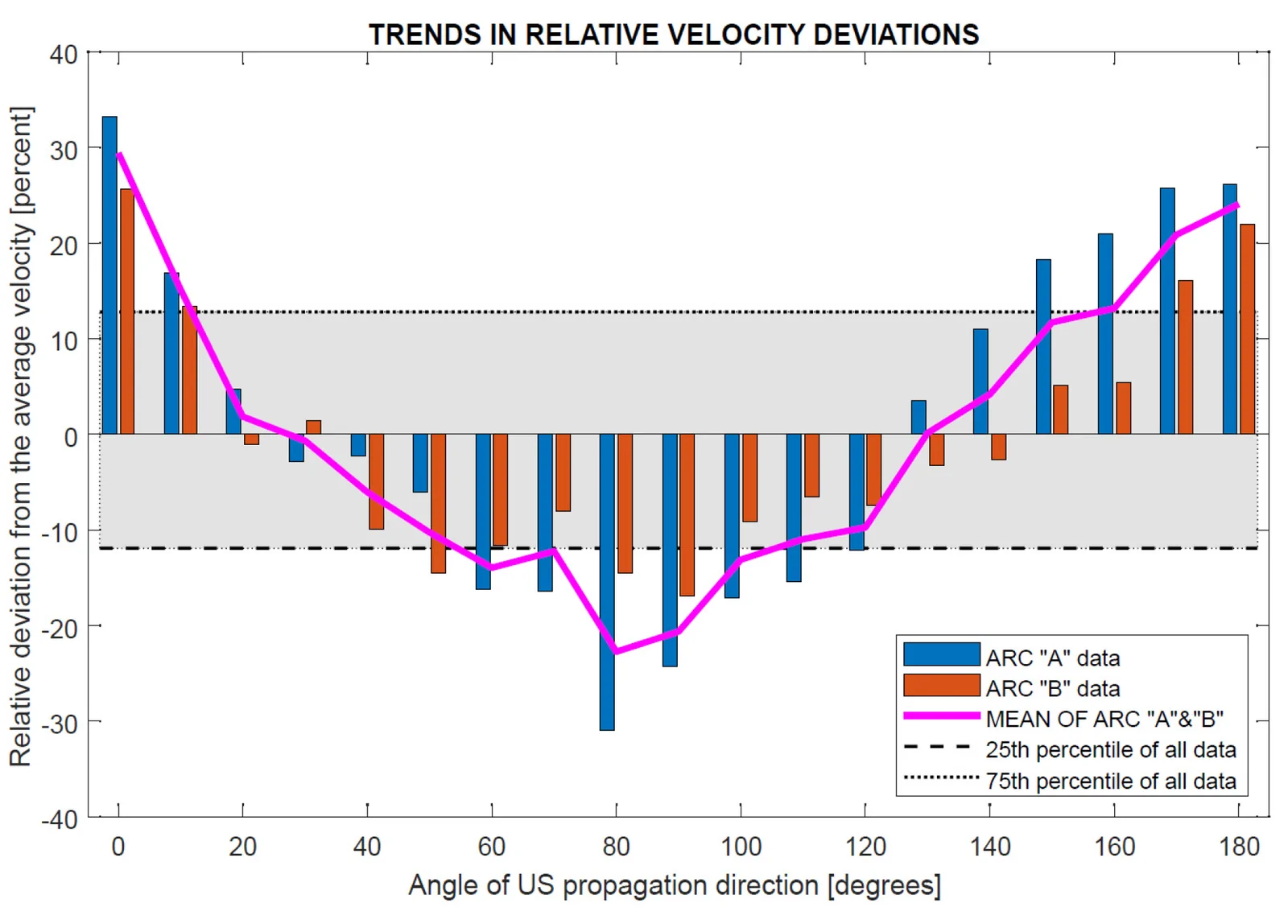
Relative deviations from average velocity in TFRC plates with different radial direction.

The relative deviation in the US velocity from the average velocity is strongly influenced by the angle of the propagation direction (Figure 11) with respect to the structural orientation of the fibers in the TFRC. An anisotropy effect is observed: the US velocity is higher for wave propagation parallel to the composite fibers and lower for propagation perpendicular to them. The relative deviation does not follow a simple linear relationship; it is modeled as a function of the angle of incidence. An appreciable loss of amplitude is observed at oblique angles; the velocity decreases as the propagation direction deviates from the structural alignment.

The values of E_1_ and E_2_, respectively, determined from the propagation velocity of the Lamb waves are 6.2 GPa for direction 0° and 5.85 GPa for 90°, values which can be correlated with those determined by static and dynamic tests (Table 4). The modulus of elasticity perpendicular to the E_3_ plane was determined by calculations, determining the compressional and shear wave velocity [62] using a code developed in MATLAB 2023b based on the FDTD method for a frequency of 1 MHz. For these values, the material parameters E_3_ = 6.26 GPa and G_13_ = 4.2 GPa were obtained.

The analysis of the US interaction with the biocomposites in this case does not aim to highlight micro-sized defects, with the flax fibers themselves having fine dimensions. The application of the technique is of interest in analyzing the distribution of fibers in the biomatrix. The speed of ultrasound (longitudinal wave) in the composite material, assumed to be QI, is calculated with the typical relation C=2L/TOF=2Lυ s for a wave propagating in an elastic medium. For the fiber content V_f_ of 35% for alkali-treated flax, a longitudinal velocity C_l_ = 2089 m/s and a transverse velocity C_t_ = 1090 m/s were obtained.

**Figure 12:**
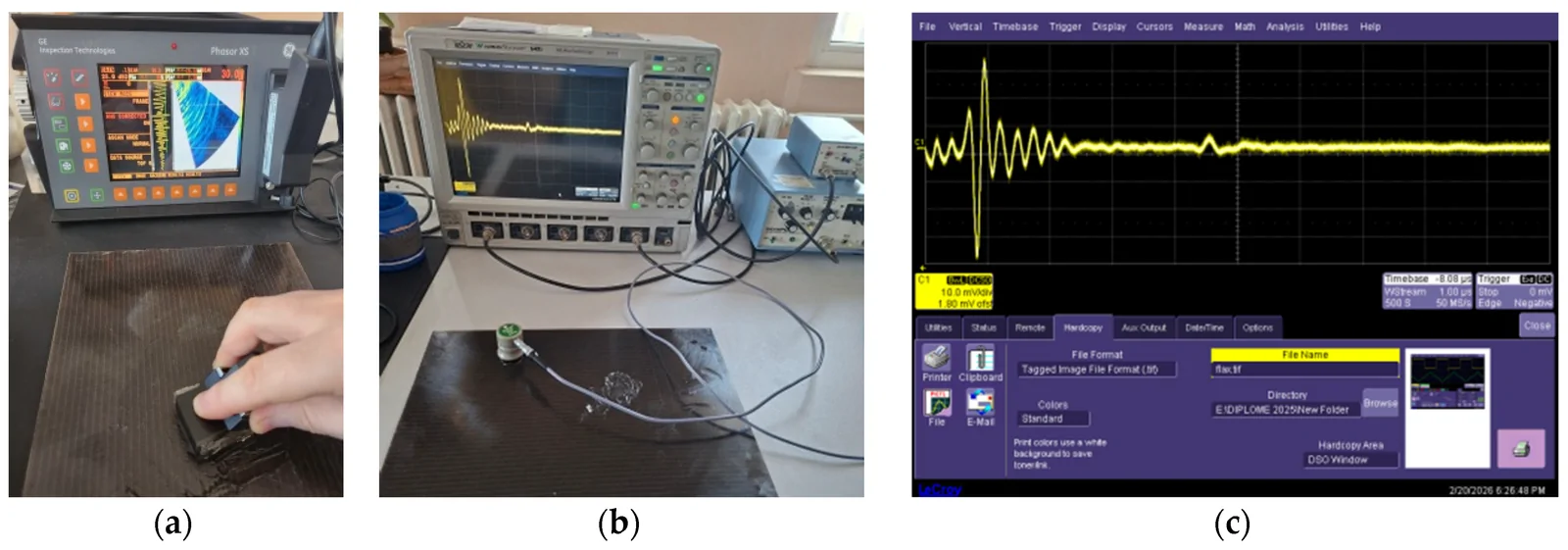
US region of interest biocomposite TFRC: (**a**) signal obtained by Phased array, S-scan; (**b**) US signal obtained by Pulse Receiver 5073PR and MB5Y-GE transducer; (**c**) US attenuation signal.

The value obtained is correlated with [63], which shows the dependence of the velocity on the percentage fiber content (V_f_) and on the material properties. The values obtained show that at a low fiber content (<40%), the voids can be negligible; an appreciable attenuation of the US signal is noted, correlated with both the fiber content and the 5 MHz frequency [62], as well as the possible existence of weak areas of fiber biocomposite adhesion (Figure 12).

Using S-scan and moving the transducer over the composite surface, each ultrasonic beam will act on a different reflector. The ultrasonic nondestructive testing method (A-scan along the angular direction as well as S-scan) of TFRC shows a fiber structure without delaminations or fiber agglomerations that generate gaps in the lamina configuration. This combines sector and line scanning, providing better coverage and image quality.

### 3.2. Tensile Test Experimental Results

Uniaxial tensile loading of the [+45/−45]_4S_ laminate generates predominantly in-plane shear stresses within the laminate, as the fibers are oriented at ±45° to the loading axis. Consequently, the fibers are not aligned with the loading direction and therefore do not effectively carry the applied load, as they would if the load was applied parallel to the fiber direction. Additionally, the measured strength is influenced by stress concentrations and edge effects typical of long and narrow specimens, which promote premature damage initiation at the specimen edges.

**Figure 13:**
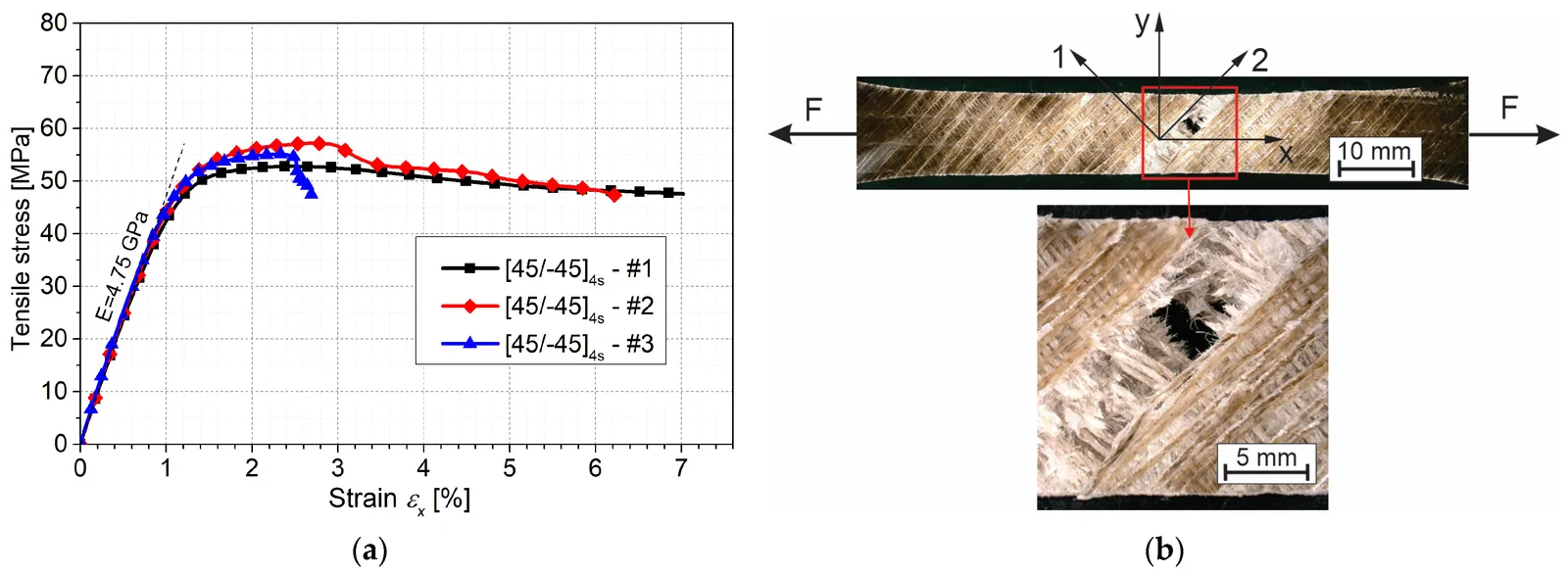
Tensile test (ASTM D638): (**a**) stress–strain diagram; (**b**) characteristic tension failure of [+45°/−45°]_4S_ flax laminate.

In Figure 13a, the characteristic diagrams obtained for the composite specimens in tension are presented. Failure is governed by matrix-dominated mechanisms, including the shear yielding of the matrix and fiber–matrix interface debonding, rather than fiber breakage, as shown in Figure 13b. The macroscopic view (top) shows a localized failure zone in the gauge section.

The detailed view (bottom) reveals damage dominated by matrix shear cracking and fiber–matrix debonding along the ±45° fiber directions. The results of the 45° off-axis tensile testing show that the tensile strength under uniaxial load is 55.2 MPa, the elastic modulus is 4.75 GPa, and the breaking strain is 5.32%, as shown in Table 4. The tensile results show high repeatability across all specimens, with closely overlapping stress–strain behavior, except for a minor deviation observed in specimen #3, and low scatter in strength and stiffness parameters, confirming a consistent tensile response of the TFRC material.

### 3.3. Compression Test Experimental Results

The results of the maximum compressive stress, total compressive displacement, and apparent elastic modulus are presented in Table 5. The test was prematurely terminated at a displacement of 6 mm, which is when the compression platen approached the fixture limit in accordance with ASTM D695.

As a result, the specimen did not reach collapse, and the true compressive strength could not be determined. Under axial compression, the [+45/−45]_4S_ laminate exhibits a shear-dominated response. The apparent elastic modulus was estimated from the ratio l/L_0_, where l_0_ = 79.4 mm is the initial specimen length and one is the measured compressive displacement.

The applied compressive stress is primarily transformed into in-plane shear stresses (τ_12_) in the material coordinate system. Since the flax fibers are not aligned with the loading direction, their contribution to axial load-bearing is limited, and the epoxy matrix carries a significant portion of the shear load.

Consequently, damage initiates in the matrix. Visible reduction in transparency in the matrix indicates shear-compressive deformation and local buckling along the specimen length (Figure 13).

Overall, the compressive results exhibit excellent repeatability, with minimal variation among specimens and closely overlapping stress–strain responses, indicating the stable, reliable compressive behavior of the TFRC material.

**Figure 14:**
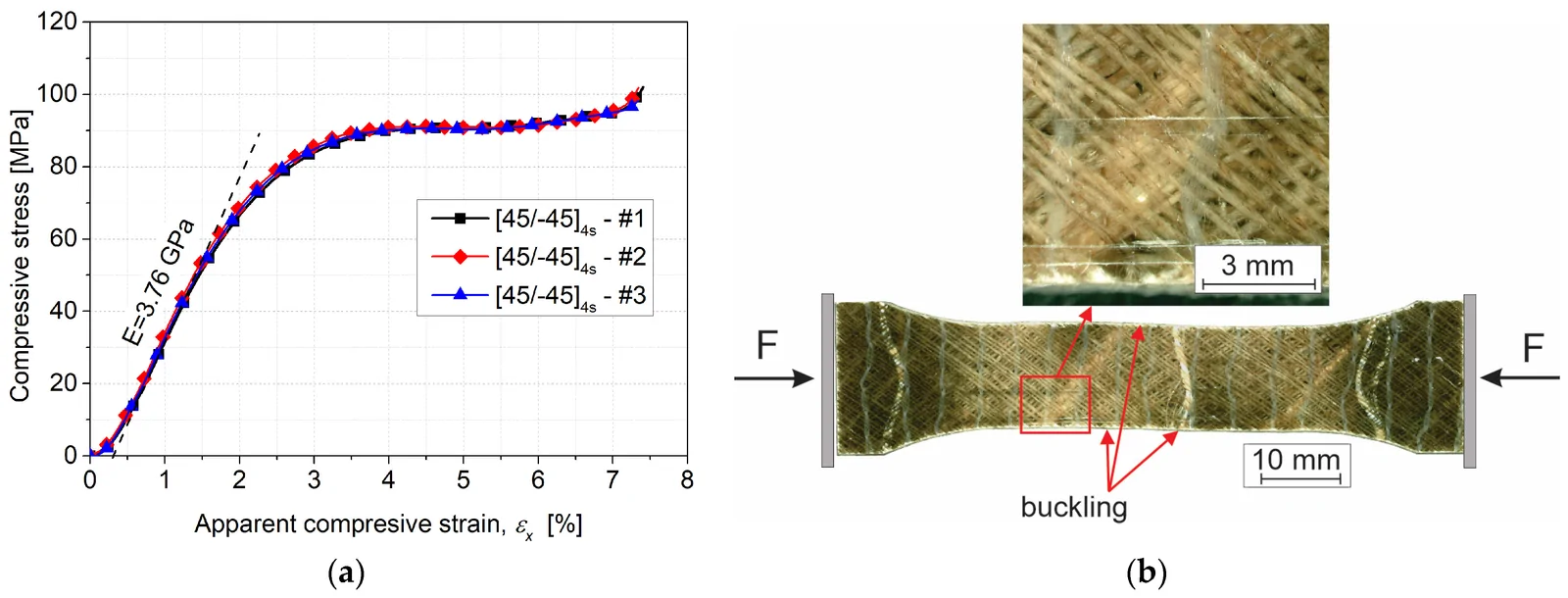
Compressive test (ASTM D695): (**a**) stress–strain diagram; (**b**) characteristic compression damage of [+45°/−45°]_4S_ flax laminate.

The compressive stress–strain response of the flax fiber-reinforced composite shows non-linear, multi-stage behavior typical of natural fiber composites (Figure 14). At low strains, a non-linear toe region appears due to specimen seating, closure of micro-voids, and initial fiber misalignment. This is followed by a quasi-linear region representing the apparent elastic response, in which both the matrix and the fibers contribute to load-carrying. With increasing strain, the curve deviates from linearity, indicating the onset of damage. A plateau-like region then develops, associated with progressive damage mechanisms such as matrix cracking, fiber–matrix debonding, and fiber microbuckling.

Despite this, the material continues to carry load due to internal stress redistribution and friction. At higher strains, the stress rises again due to progressive compaction of the material.

The [+45/−45]_4S_ flax composite exhibits shear-dominated behavior in both tensile and compressive loading due to the off-axis fiber orientation, but the damage evolution and overall response differ significantly. Under tensile loading, the material exhibits a lower strength (55.2 MPa) than under compression. The applied load is transformed into in-plane shear, limiting the contribution of fibers and leading to matrix-dominated failure through shear cracking and fiber–matrix debonding. Failure is localized and occurs relatively early, with no significant fiber breakage. In contrast, under compressive loading, the composite reaches a higher apparent strength (99.1 MPa), although the test was displacement-limited.

The response is more progressive, with gradual damage development including matrix deformation, debonding, and local buckling. Unlike tension, the material continues to carry load after damage initiation due to stress redistribution and compaction effects. Overall, tensile loading leads to earlier and more localized failure, whereas compressive loading promotes gradual damage accumulation and sustained load-carrying capacity.

In ref. [63], the engineering elastic constants, Young modulus, Poisson’s ratio, Shear modulus, and density of TFRCs were identified using the finite element updating method (FEMU), as given in Table 6.

### 3.4. Dynamic Mechanical Analysis (DMA)

Cross-ply and QI flax composites are materials with potential special properties (a balance between weight and strength/stiffness). These are obtained by overlapping layers with fibers oriented in different directions (i.e., 0°; 90°; ±45°), aiming to obtain superior performance [64]. QI laminates, in the case of TFRC, offer uniform in-plane strength and good fatigue resistance, making them competitive with GFRP in some applications. However, experiments show that they are susceptible to impact damage when the damage forms a fan-like opening.

The structure of these composites requires analysis of the matrix, the fibers, and the fiber–matrix interactions that define the thermomechanical performance. In DMA analysis, the components of the complex elastic modulus are a phase vector that incorporates the elastic component (storage modulus, E′) that quantifies the material’s ability to store elastic energy as well as the viscous component (loss modulus, E″) that quantifies the material’s ability to absorb energy.

(2) $$E^{*}\left(\omega \right)=E^{\prime }\left(\omega \right)+jE"\left(\omega \right),\;j^{2}\;=\;-1$$

For materials with negligible damping, the storage modulus is equivalent to Young’s modulus. The loss factor tg δ, as the ratio between the two quantities, represents the damping capacity (loss factor) $\tan\delta =\frac{E^{\prime \prime }(\omega )}{E^{\prime }(\omega )}$. To study the dynamic behavior of TFRC, DMA analysis was performed on four specimens at the equipment’s test frequencies over a temperature range of [27 ÷ 220] °C.

Using a DMA 242, the principal mechanical properties of the composite, as well as the glass transition temperature Tg, were determined, with the results presented in Table 7. Overall, the DMA results show consistent trends across four specimens at both frequencies, with only moderate scatter in measured properties, confirming a reproducible viscoelastic response of the TFRC material.

**Figure 15:**
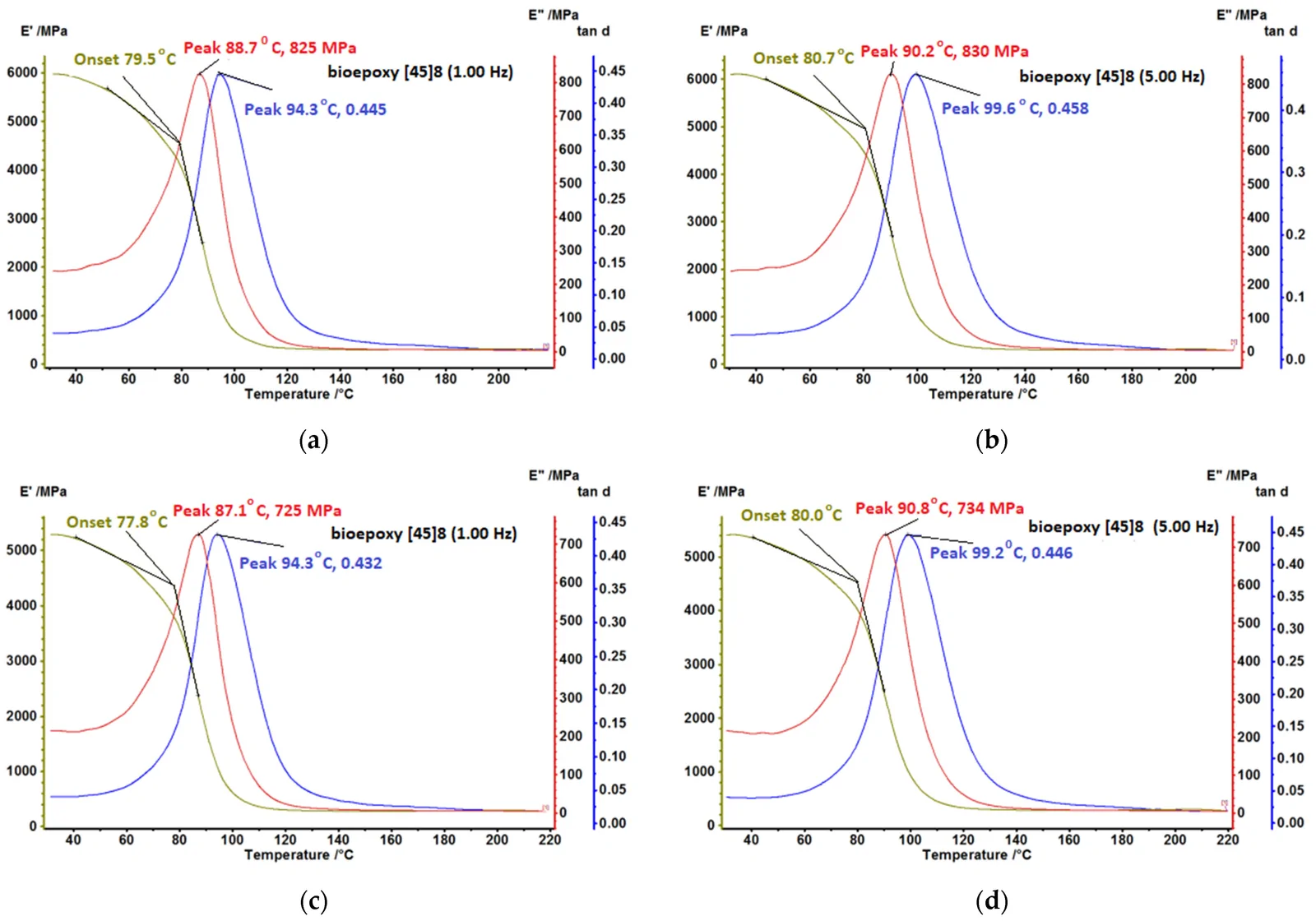
DMA test of TFRC at frequency 1 Hz and 5 Hz: (**a**,**b**) sample 02; (**c**,**d**) sample 03.

Figure 15 shows the test results for the specimens marked 02 and 03, respectively, in Table 7. Samples 01 and 02 have the length along the e_1_ axis (Figure 1b) of the biocomposite plate. Samples 03 and 04 are for the direction inclined by 15 degrees to the e_1_ axis. DMA tests performed on cut samples with random length relative to the ±45° direction aimed to elucidate the relationship between the failure mechanisms presented in [56], in which the [±45°]_4S_ laminates exhibit improved in-plane shear strength, and the phase vector incorporates the elastic components E′ and the storage modulus E″.

DMA analysis of TFRC shows that as the temperature increases, the loss modulus E′ respects the three phase transition zones at the vibration frequencies. For a fiber volume of 35%, the experimentally obtained data show that up to a temperature of 50 °C, the modulus of elasticity is maintained in a slow variation of about 300 MPa, and then presents a more pronounced decrease up to 80 °C, followed by a “violent” fluctuation over a temperature range between [80 ÷ 100] °C.

At this temperature, the glass transition occurs—Tg = 79.5 °C at an oscillation frequency of 1 Hz (Figure 15a) and Tg = 80.7 °C at an oscillation frequency of 5 Hz (Figure 15b)—the temperatures at which the material loses its structural rigidity. In relation to the fiber direction, the temperature difference at the glass transition does not differ significantly with frequency.

It is observed that the variation in E′ of the biocomposites is greater in the glassy region, and then at a temperature of 80 °C it decreases due to the reinforcement fibers losing their rigidity at high temperatures. This indicates that temperature plays a significant role in the interfacial properties and adhesion of the bio-based resin, with direct implications for mechanical performance.

In the range [110 ÷ 180] °C, the biopolymer in-matrix system has a small but balanced E′ modulus due to the mobility of the polymer chain network. The strength of the adhesive is closely related to temperature; once the temperature approaches the material’s glass transition temperature (Tg), the rigid state disappears, leading to a decrease in strength. Tg represents the critical limit for proper functioning of the composite material.

Understanding these factors can enable the successful production and use of a bio-based flax composite with a [45°]_4S_ stacking sequence, achieving high yield, high performance, and sustainability. In relation to the storage modulus (E″), it can be observed that due to the presence of fibers with [±45°]_4S_ orientation, the flax composites in the glassy region have an increased storage modulus. E″ provides information on the amount of energy dissipated as heat per oscillation cycle. As the material transitions from the glassy to the “highly elastic” state, the properties of the biopolymer become predominant. Analysis of the graph shows that TFRC has a narrower E″ curve width compared to polymer composites.

Advanced characterization provides data for understanding the performance/use limits of bio-epoxy composites. Regarding the packing mode, the presence of fibers with [45°]_4S_ orientation results in an increased storage modulus for flax composites in the glassy region. The loss factor (tanδ) provides information about the interaction between the fibers and the matrix and measures the impact properties of the TFRC.

Tanδ for [±45°]_4S_ has a value in the range [0.430 ÷ 0.452] for a frequency of 1 Hz, and, respectively [0.440 ÷ 0.465], for a frequency of 5 Hz (varies depending on the oscillation frequency) because the bio-epoxy resin has a high molecular mobility (demonstrated by the peak value), which is stopped by incorporating the fibers into the given structure. At the maximum value, corresponding to the glass transition temperature (Tg), the peak of the loss coefficient (tanδ) represents the temperature at which chemical changes occur in the structure of lignin and cellulose. As the oscillation frequency increases, the response time to the load decreases, and the ratio of dissipated to stored energy increases. This establishes strong adhesion of the fibers to the matrix, resulting in uniformly distributed tension. The lower damping curve for TFRC compared to carbon-based composites indicates that the material can absorb more energy and becomes stiffer at a slightly higher temperature. Tanδ curves shift slightly towards higher temperatures as the frequency increases.

**Figure 16:**
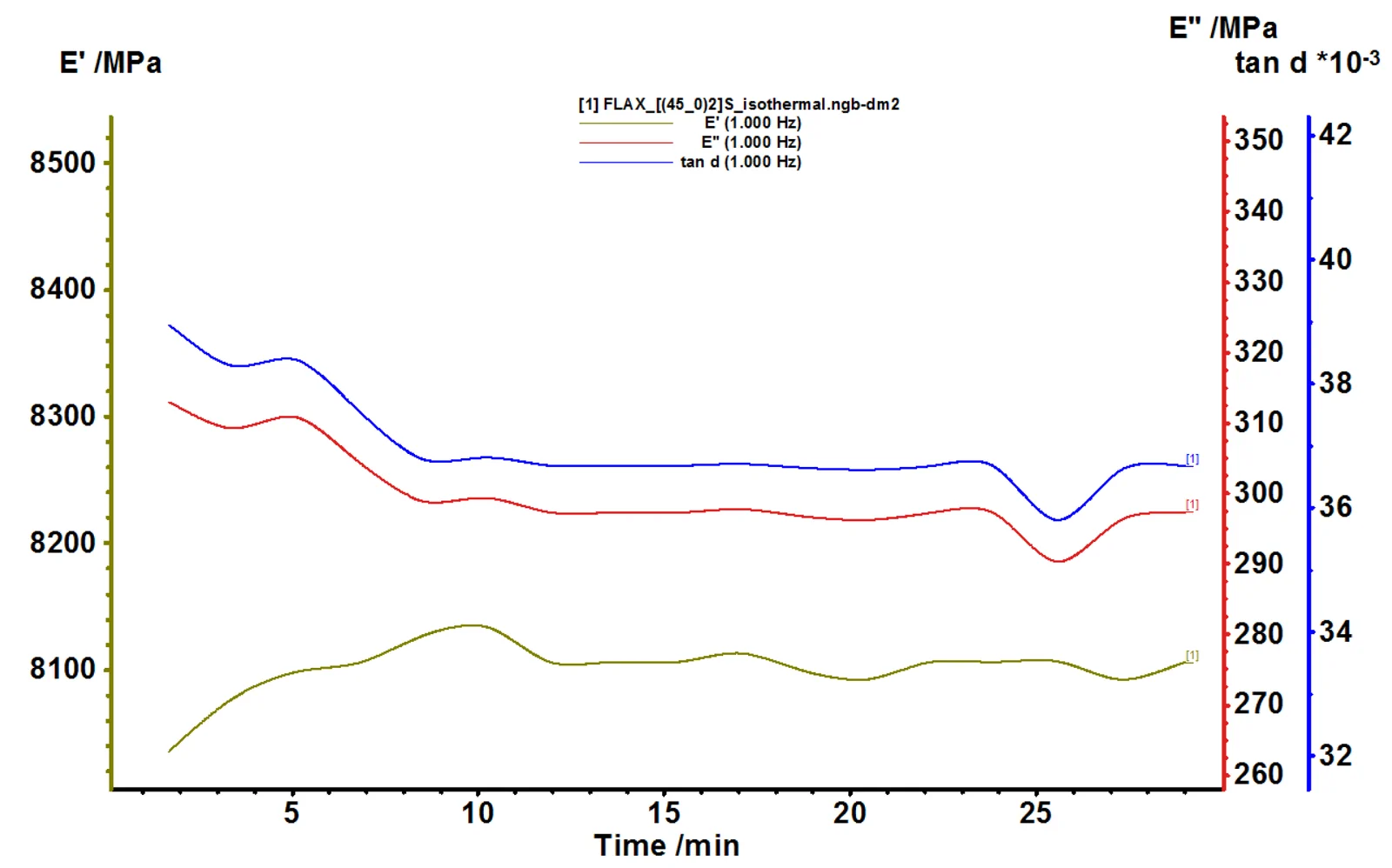
DMA test of TFRC at frequency 1 Hz for sample 02.

It can be concluded that tanδ is a critical measure of the interface between the flax fibers and the matrix and measures the impact properties of TFRC. Figure 16 shows the heating profile at a constant temperature of 45 °C for one of the samples (sample 02) at a frequency of 1 Hz.

It can be observed that for a temperature of 45 °C, in a time interval of 30 min, the storage modulus (E′) has, after 10 min, a slight increase from 8039 to 8136 MPa (an increase of 1.2%), which can be attributed to the viscosity of GreenPoxy56 molecular structure and the physical properties of SD7561 hardener. This trend then decreases to 8091 MPa (a 0.55% decrease), followed by relative stability around this plateau value, which is important for structural stability.

The increase in E′ occurs at the same time as the loss modulus (E″) decreases from 312 to 290 MPa (a 7% decrease), which shows that over time at a temperature of 45 °C, there is an improvement in interfacial adhesion and better and more uniform stress transfer (in practice, internal friction is reduced). During the 30 min period, the loss factor (tg δ), defined as the ratio of E″ to E′, decreases by 7.9% (indicating a higher cross-linking density) over the 10 min interval, after which it plateaus at 36.8 × 10^−3^.

Chemical treatments (performed by the flax fiber manufacturer) (i.e., alkali, maleic anhydride) can further improve the fiber–matrix interface, leading to lower damping values and superior mechanical properties.

### 3.5. Computational Aspects

**Figure 17:**
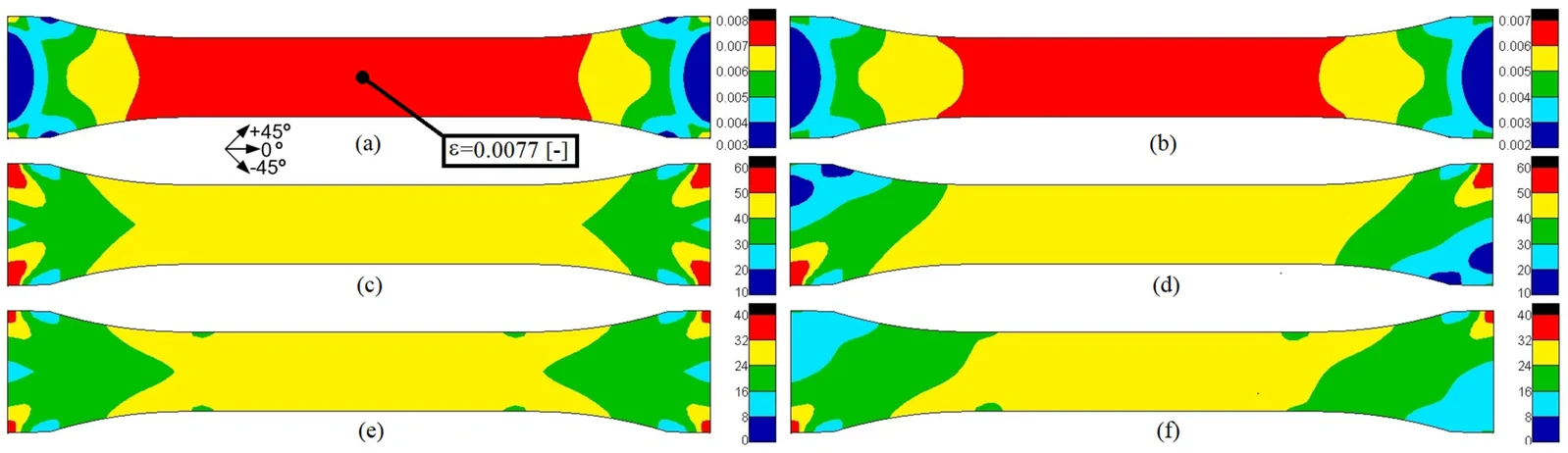
FEA result plots for tension: (**a**) maximum principal strain [-]; (**b**) maximum shear strain [-]; (**c**) maximum principal stress [MPa], maximum between all plies; (**d**) maximum principal stress [MPa] for a +45° ply; (**e**) maximum shear stress [MPa], maximum between all plies; (**f**) maximum shear stress [MPa] for a +45° ply.

**Figure 18:**
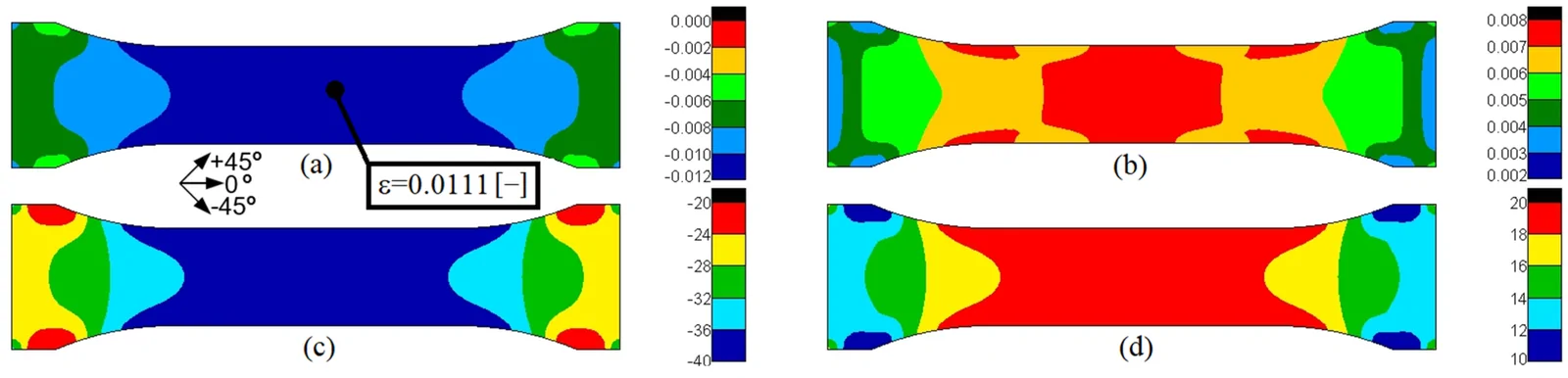
FEA result plots for compression: (**a**) minimum principal strain [-]; (**b**) maximum shear strain [-]; (**c**) minimum principal stress [MPa], minimum between all plies; (**d**) maximum shear stress [MPa], maximum between all plies.

The numerical simulation results presented in Figure 17 and Figure 18 indicate that the fiber orientation (stress plots for plies at −45° fiber orientation are symmetrical to +45°) leads to localized stress and strain concentrations near the specimen edges. Still, these effects cancel out toward the center region, where the stress and strain fields become more uniform due to load redistribution among the plies. To enable comparison with experimental results, strain values (minimum/maximum principal strain) were extracted from the model central region (uniform loading area). For tension, the difference between FEM and test results is 3.8% (FEM strain 0.0077, test strain 0.0079). For compression, the difference between FEM and test results is 0.9% (FEM strain 0.0111, test strain 0.0110). For both cases, a good agreement between numerical and experimental results is observed. Small deviations in fiber position, alignment, and other localized defects in the plies of the real laminate can explain the differences.

In contrast, the FE model assumes a perfect material. Also, the loading mechanism accounts for some of the differences in results. Under tensile loading, the fibers primarily carry the load, whereas under compression, matrix yielding dominates the behavior and becomes the primary driver. As stated above, the FE model is based on the linear static analysis (Solution 101 in MSC Nastran) and predicts stresses and strains under the assumption of linear elasticity.

Based on the results presented in this chapter, the FE model validates the stiffness of flax fiber-reinforced composites measured by mechanical tests below the yielding threshold.

The study of composites with cross-laminated laminates is analyzed in the specialized literature given their properties during use engineering instructions [65]. Double layers in folds were studied by Tsai for different angular orientations θ of the fibers, aiming to reduce the bending–twisting coupling [66]. In refs. [67,68], the results of the macromechanical analysis used to determine the off-axis stiffness and strength properties for unidirectional orthotropic blades are presented. Combined tests on the TFRC composite in the stacking configuration [±45°]_4S_ followed the analysis of tensile-compressive behavior to increase the structure’s efficiency by maximizing weight efficiency. The structure was obtained by imposing stacking sequences symmetrical with the orientation of the UD fibers in the ply (θ = 45°), after a balanced design with layers having double folds, analyzing some of the composites used in aeronautics [69]. Optical analysis of flax fibers (FF) revealed an increase in thread compactness, deformation of the cross-section, and a significant reduction in porosity. This was also highlighted in mechanical tests: under compression load, the composite reached a higher apparent strength (99.1 MPa), although the test was limited by displacement. Under tensile load, the material shows a lower strength (55.2 MPa) than in compression. The capacity of double folds in a layer at [±45°] to limit damage to localized areas has been studied theoretically [62], evaluating failure theories based on physical principles (Hashin, Camanho, Puck, LaRCo5) to predict defect initiation in fiber laminates focusing on failure mechanisms [68,70]. Monitoring the structural condition of flax fiber biocomposite involves the real-time analysis of the damage and internal stress of the constituent components [71], providing data for the characterization of UD biocomposites. The FE models validate the results of mechanical tests up to the yield point. The applied load in FEA is chosen in the linear-elastic region and is adopted for operational loads that remain below the onset of damage. It was found that for FFs not aligned with the loading direction, their contribution to the axial load-bearing capacity is limited, and the epoxy matrix supports a significant part of the shear load. DMA analysis shows consistent trends across the four specimens at 1 Hz and 5 Hz, with only a moderate spread in the measured properties, confirming a reproducible viscoelastic response of the bicomposite. The increase in the elastic modulus (E′) occurs at the same time as the 7% decrease in the loss modulus (E″), which shows that, over time, at a temperature of 45 °C, there is an improvement in the interfacial adhesion, as well as a better and more uniform stress transfer. The structural changes in the fibers, resulting from the alkaline treatment, are associated with a high level of impregnation and compaction in the epoxy resin. DMA analysis by the peak of the loss tangent (tanδ) at its maximum value in the range [0.440–0.465] at 5 Hz demonstrates that the alkali treatment facilitates the efficient transfer of mechanical loads from the fibers to the fiber–matrix interface. This improves the fiber–matrix interface, leading to lower damping and superior mechanical properties, with flax naturally acting as a built-in shock absorber. For the determination of the US propagation velocity, A-scan and S-scan images showed a fibrous structure without delaminations. The application of the US technique aimed to analyze the distribution of fibers in the biomatrix, without delaminations or fiber agglomerations that generate voids for the lamina configuration. The results were correlated with those from the mechanical testing: under compression load, the composite reached a higher apparent strength (99.1 MPa), although the test was limited by displacement. The mechanical and elastic properties of biocomposites depend not only on the resin but also on the nature of the reinforcement, the stacking sequences, and the properties of the laminae. The interfacial adhesion also influences the mechanical behavior of biocomposites. The analysis of the mechanical properties of TFRC in the given structure (tensile behavior) shows good repeatability, with superimposed stress–strain behavior. TFRC has low dispersion of the stiffness and strength parameters.

Although environmental standards are forcing the industry to adopt environmentally friendly materials, fiber-reinforced polymer (FRP) still dominates. With the increasing global attention to the sustainability of composite materials, the momentum towards bio-epoxy/natural fiber composites is growing as a solution to problems related to unsustainable raw material production. As an ecological and environmental alternative, bicomposites with flax fiber reinforcement have gained attention due to their recyclability, low cost, high specific strength and stiffness, and low density [72], being able to compete with glass fibers. Knowledge and prediction of the stress–strain states of bio-epoxy composites FRPC are important when analyzing the possibility of FRP replacement. Exploiting the possibility of appropriate use of the biocomposite TFRC and analyzing the behavior of the constituents can potentially ensure improved applications of semi-structural components. FRPC can contribute to reducing the ecological footprint and maintaining sustainability, leading to reduced material waste [73]. However, once they reach the end of their useful life, ecological sustainability, ironically, still has a long way to go. Integration into the circular economy model [57] requires adapting sustainable practices not only for the raw material but also for the production stages.

## 4. Conclusions

The present study analyzes customized flax-reinforced composites (TFRC) with a stacking sequence [±45°]_4S_, designed to achieve high damage tolerance, which is necessary to increase the strength-to-weight ratio. The lamellae are composed of two unidirectional, weakly twisted layers of alkali-treated flax yarns, oriented at ±45°, and reinforced by Greenpoxy56/SD7651 bio-resin-impregnated seams.

By exploiting an industrial textile technique that uses lightly twisted UD linen yarns, arranged in a specific lamination sequence, a material with low weight and optimized mechanical response has been successfully developed.

The main findings in the analysis of the TFRC structure are as follows:

- Improved sustainable performance—TFRC with a fiber volume fraction of 35% demonstrates properties comparable to semi-structural composites while maintaining a lower density (1254 kg/m^3^), confirming its potential for weight-critical applications in civil and aerospace engineering.
- Progressive damage behavior—[±45°]_4S_ stacking sequences produce a matrix-dominated in-plane shear response. This finding in tensile/compression testing confirms that the fiber orientation strategy in both the laminates and the composite laminates effectively promotes progressive damage and high strength compared to the failure modes typical of conventional rigid laminates.
- Thermomechanical stability and interfacial integrity and homogeneity—DMA tests confirmed that TFRC maintains a high storage modulus in the glassy region, with a stable plateau at critical temperatures (e.g., 45 °C) essential for structural applications where consistent mechanical properties are required under different environmental conditions. The relative homogeneity of the fiber distribution was confirmed, and the importance of the alkaline treatment of the fibers, which reduced their diameter, ensuring compactness and significantly reducing porosity, was confirmed. An efficient charge transfer at the fiber–matrix interface was obtained
- Validation by simulation and experiment—experimental results in tension and compression, as well as those from simulation, confirm that the classical laminate theory, applied in the case of fiber orientation at [±45°], correctly predicts the elastic stiffness response below the yield threshold

Overall, the research improves the understanding of the behavior of the customized arrangement of flax fibers in a [±45°]_4S_ configuration. The structure provides the necessary solid reinforcement and damage tolerance for specialized engineering applications, enabling the large-scale support and deployment of renewable, bio-based resources in advanced industrial sectors without compromising mechanical safety or performance.

### Perspectives

In-based composites with stacking sequence [±45°]_4S_ show promising mechanical properties for research in critical areas, which is why additional application-specific research could be performed to ensure long-term structural reliability.

A special aspect of the investigations is the analysis of the long-term effects of aging on the constituents of the composite.

## Figures and Tables

**Table 1:** Properties of the elementary flax fiber [44,45].

Property	Parameter	Values	Units
chemical	cellulose	64.1–75.0	wt%
	hemicellulose	10.37–20.6	wt%
	pectin	2.0–5.0	wt%
	lignin	2.2	wt%
	waxes	3.5	wt%
	proteins	2.0–5.0	wt%
mechanical	elongation at break	1.2–10	%
	Young’s modulus	24–80	GPa
	tensile strength	88–1600	MPa
physical	absorption regain at 65% r.h., 20 °C	7.0	%
	microfibrillar angle	5–10	degrees
	density	1.40–1.54	g/cm^3^
	thickness of wall	20.0	µm
	width of lumen	6.42	µm
	fiber thickness	38.0	µm
	fiber length	65.0	mm

**Table 2:** Structural characteristics of the flax yarns.

Characteristic	Symbol	Values		Units
		Dry Yarns	Yarns in Matrix	
linear density	T_tex_	100	42.5	g/km
yarn count	Nm	10	23.5	m/g
diameter	D	0.3965	0.3176	mm
area	A	0.1123	0.0793	mm^2^
twist angle	β	12	-	degrees
twist coefficient	α_m_	80	-	-
yarn twist	T	250	-	twists·m^−1^
yarn constant	C	1.254	1.54	-
thickness two plies [±45°]		0.660	-	mm/layer
TFRC [±45°]_4S_ thickness		-	5.38	mm

**Table 3:** Tensile test of flax yarns and epoxy resin.

Material Type	Breaking Force F_max_ [N]	Tensile Strength σ [MPa]	Young’s Modulus E [GPa]	Elongation at Break ε_B_ [%]
flax yarns	37.5 (±4.3)	345 (±38)	25.2 (±1.7)	1.920 (±0.21)
epoxy resin	2803	71.2	3.2	4.91

**Table 4:** Tensile strength and breaking strain for [+45°/−45°]_4S_ TFRC specimens, n = 3.

TFRC [+45/−45]_4S_	Tensile Strength [MPa]	Elastic Modulus [GPa]	Breaking Strain ε_B_ (%)
Specimen#1	57.3	4.58	7.02
Specimem#2	55.2	4.78	6.22
Specimen#3	53.1	4.91	2.71
Average	55.2	4.75	5.32
St. dev.	2.14	0.16	2.29
Var. coeff. (%)	3.88	3.4	43.1

**Table 5:** Compressive stress, absolute compression and apparent elastic modulus for TFRC specimens, n = 3.

TFRC [+45/−45]_4S_	Max. Compressive Stress [MPa]	Absolute Compression (mm)	Apparent Elastic Modulus [GPa]
Specimen#1	100.8	5.854	3.75
Specimem#2	99.06	5.829	3.81
Specimen#3	97.08	5.826	3.72
Average	99.11	5.836	3.76
St. dev.	1.44	0.015	0.0458
Var. coeff. (%)	3.44	0.263	1.22

**Table 6:** Transversely isotropic material properties of flax fiber–epoxy UD composite obtained by FEMU method [63].

E_1_ [GPa]	E_2_ [MPa]	ν_12_ = ν_13_	ν_23_	G_12_ = G_13_ [GPa]	G_23_ [GPa]	Density [kg/m^3^]
22.56	1.376	0.12	0.4	1.477	1.18	1254

**Table 7:** Values of TFRC samples’ storage modulus E′; loss modulus E″; glass transition temperature Tg; maximum value of tanδ.

Sample V_f_ = 0.35 [%]	Length [mm]	Width [mm]	Average Thickness [mm]	E′_max_ [MPa]		E″_max_ [MPa]		Tg [°C]		tanδ max	
				1 Hz	5 Hz	1 Hz	5 Hz	1 Hz	5 Hz	1 Hz	5 Hz
01	50.70	10.51	5.50	6072	6152	815	827	80.8	82.8	0.452	0.465
02	50.75	10.42	5.38	6000	6252	825	830	79.5	80.7	0.445	0.458
03	50.72	10.49	5.42	5248	5410	725	734	77.8	80.0	0.432	0.446
04	50.25	10.46	5.40	5275	5400	732	743	80.4	82.2	0.43	0.44

## Data Availability

The original contributions presented in this study are included in the article. Further inquiries can be directed to the corresponding authors.
